# Serotype distribution of invasive and non-invasive pneumococcal disease in children ≤5 years of age following the introduction of 10- and 13-valent pneumococcal conjugate vaccines in infant national immunization programs: a systematic literature review

**DOI:** 10.3389/fpubh.2025.1544359

**Published:** 2025-05-30

**Authors:** Patricia Izurieta, Mohammad AbdelGhany, Dorota Borys

**Affiliations:** ^1^Vaccines R&D/Infectious Disease, GSK, Wavre, Belgium; ^2^Vaccines Institute of Global Health, GSK, Siena, Italy

**Keywords:** acute otitis media, children, community-acquired pneumonia, invasive pneumococcal disease, pneumococcal conjugate vaccine, serotype distribution, *Streptococcus pneumoniae*

## Abstract

**Introduction:**

Widespread implementation of pneumococcal conjugate vaccines (PCVs)—namely the 7-valent PCV (PCV7), 10-valent pneumococcal non-typeable *Haemophilus influenzae* protein D conjugate vaccine (PHiD-CV), and 13-valent PCV (PCV13)—in infant national immunization programs has reduced pneumococcal diseases in children, including invasive pneumococcal disease (IPD), acute otitis media (AOM), and community-acquired pneumonia (CAP). However, as the use of PCV impacts pneumococcal epidemiology, identifying the serotypes associated with remaining disease is crucial to guide future vaccination strategies for this population.

**Methods:**

We systematically searched the literature for observational studies (2006–2020) on pneumococcal serotype distribution in IPD, AOM, and CAP among ≤5-year-old children post-PCV introduction. Serotype-specific pooled percentage averages were calculated by post-PCV period (post-PCV7 or pooled post-PHiD-CV/PCV13), or by PCV product (PHiD-CV or PCV13) to determine the contribution of each serotype to a certain clinical manifestation.

**Results:**

Our analysis of 86 studies (47 on IPD, 30 on AOM, and 9 on CAP) shows continued reporting of several vaccine serotypes in all clinical manifestations post-PHiD-CV/PCV13, particularly serotypes 19A, 3, and 1. In PCV13 settings, serotype 19A reporting was reduced but still prevalent compared to PHiD-CV settings. Predominant non-PCV13 serotypes varied by clinical manifestation.

**Conclusion:**

Post-PCV implementation, pneumococcal epidemiology in children is intricate. The persistence of some vaccine serotypes, variations across clinical manifestations, rising antimicrobial resistance, and other factors highlight the need for new vaccine technologies providing enhanced and broader protection to children.

## 1 Introduction

*Streptococcus pneumoniae* (Spn) is a major bacterial cause of a wide range of infections, which can be broadly grouped into invasive pneumococcal diseases (IPD), including meningitis and septicemia, and non-invasive diseases, such as acute otitis media (AOM) and community-acquired pneumonia (CAP) (1–5). Non-invasive forms of these infections may become invasive (e.g., when CAP is accompanied by bacteremia) (6, 7). Young children (≤5 years of age), older adults (≥65 years of age), and those with underlying medical conditions are at increased risk of pneumococcal infections (1, 6, 8, 9).

Although all of the at least 100 identified Spn serotypes are theoretically capable of causing disease (10), only a subset is responsible for most pneumococcal infections. The prevalence and distribution of (disease-causing) serotypes vary by age, geographical location, clinical manifestation, and antibiotic use (8, 11–17). Nonetheless, the major driver of changes in pneumococcal epidemiology over time has been the global implementation of pneumococcal conjugate vaccines (PCVs) which has led to serotype replacement (18). This occurs as serotypes included in the PCV decline following vaccination, allowing non-vaccine serotypes to expand—a process that typically becomes evident around 4 years after vaccine introduction (19). The 7-valent PCV (PCV7; *Prevenar/Prevnar*, Pfizer Inc.) (which contains serotypes 4, 6B, 9 V, 14, 18C, 19F, and 23F) was the first approved PCV and was included in many infant national immunization programs (NIPs) between 2006 and 2008 (20). The pneumococcal non-typeable *Haemophilus influenzae* protein D conjugate vaccine (PHiD-CV, *Synflorix*, GSK) and 13-valent PCV (PCV13, *Prevenar 13/Prevnar 13*, Pfizer Inc.) replaced PCV7 in NIPs since 2009 and provided coverage for additional serotypes (PHiD-CV contains all PCV7 serotypes + 1, 5, and 7F; PCV13 contains all PHiD-CV serotypes + 3, 6A, and 19A) (11). Starting from 2015, the World Health Organization (WHO) estimated PCV coverage in 1-year-old children in high-income countries to be ≥80% (21). In low-and middle-income countries, PCV coverage has been lower, with estimates of 28 to 60% in 2015, slowly increasing in subsequent years (21).

Despite the considerable reduction in disease burden by infant vaccination with PHiD-CV and PCV13 (22–27), Spn remains a major cause of morbidity and mortality in children (28). Monitoring the evolution of pneumococcal epidemiology to evaluate the (long-term) effectiveness of vaccines and vaccination strategies is critical. Spn serotype distribution is best characterized for IPD, as it is a reportable disease, and serotyping is routinely conducted as part of many IPD surveillance programs (11). However, IPD-focused surveillance strategies may not reflect the true prevalence of pneumococcal serotypes. CAP (mainly in adults) and AOM (in children) represent the highest proportion of the overall pneumococcal disease burden (8, 29), but data on serotypes causing these manifestations are scarcer. This is because their diagnosis is often based on clinical presentation without routine collection of biological specimens, as obtaining samples for etiological diagnosis can be challenging and the conventional diagnostic tools for CAP exhibit limited sensitivity (11, 30–33).

The objective of this systematic literature review (SLR) was to summarize the global evidence from published observational studies on the serotype distribution in both invasive and non-invasive pneumococcal disease among children ≤5 years of age after the implementation of PHiD-CV and PCV13, compared to the post-PCV7 era. With the recent introduction of the 15-valent (PCV15, *Vaxneuvance*, Merck Sharp & Dohme LLC, a subsidiary of Merck & Co., Inc. [MSD]) and 20-valent (PCV20, *Prevenar 20/Prevnar 20*, Pfizer Inc.) PCVs in infant NIPs, whose epidemiological impact is still to be determined, along with ongoing pneumococcal vaccine development, we aimed to better understand the impact of PHiD-CV and PCV13—both widely implemented for many years—on the pneumococcal epidemiology. Specifically, we focused on the contribution of individual serotypes to remaining IPD, CAP, and AOM in children.

## 2 Methods

This analysis is part of a larger SLR that aimed to assess the effect of widespread PHiD-CV/PCV13 usage in infants on the serotype distribution in remaining invasive and non-invasive pneumococcal disease in children aged ≤5 years and adults aged ≥65 years. This manuscript presents the results in children. Results in older adults are summarized separately (34). The SLR was conducted in accordance with its protocol and with the Preferred Reporting Items for Systematic reviews and Meta-Analyses (PRISMA) guidelines (35).

### 2.1 Systematic search strategy

PubMed and EMBASE were searched for articles published from 1 January 2006 to 31 December 2020 on pneumococcal serotype distribution in IPD, CAP, or AOM after infant uptake of PCV7, PHiD-CV, or PCV13. The 2006 cutoff was chosen because several countries (including the United States, Canada, Australia, the United Kingdom, France, Belgium, Germany, and Italy) had already universally introduced PCV7 by 2006. We used a 2020 cutoff to avoid the immediate and rebound effects of the coronavirus disease 2019 (COVID-19) pandemic, an exceptional event that significantly disrupted surveillance systems, vaccination programs, medical care access, and disease trends for several years (36–39). Post-PHiD-CV/PCV13 serotyping data were of primary interest; data collected after PCV7 implementation were included to assess changes in serotype distribution before and after PHiD-CV/PCV13 uptake. A broad search strategy was applied using combinations of search strings, consisting of terms for Spn serotypes, PCVs, and pneumococcal diseases (Supplementary methods).

### 2.2 Eligibility criteria and study selection

All eligibility criteria were determined upfront in the protocol and were applied at screening and the data extraction phase.

As many studies on IPD were expected, eligible IPD studies were first limited to SLRs/meta-analyses. Since only 2 SLRs were retrieved, both with several overlapping studies among their respective datasets (40, 41), eligibility was expanded, as predefined, to include the most recent observational studies published between 2018 and 2020. Only studies reporting serotyping data of at least 30 isolates obtained from sterile sites were included (42).

For AOM and CAP, only observational studies were included. AOM was to be defined by clinical diagnosis (the presence of inflammation of the middle ear, associated with effusion, accompanied by a rapid onset of symptoms, and signs of an ear infection) (43, 44). Only studies that reported serotyping data of at least 30 isolates from middle ear fluid samples were included.

CAP was to be defined as pneumonia acquired outside of the hospital (45), and studies reporting serotyping data on samples obtained from either sterile sites (aligned with definition of invasive CAP) or non-sterile sites (non-invasive CAP) were included. Given the overall limited number of studies on this outcome in children aged ≤5 years, no distinction was made between non-invasive and invasive CAP, and the minimal number of reported serotyped isolates was set at 20 instead of 30.

Further details on inclusion/exclusion criteria and study selection workflow are provided in Supplementary methods, Supplementary Tables S1–S3.

### 2.3 Data extraction and analysis

The contribution of each serotype to a certain clinical manifestation was determined by calculating pooled percentage averages for each serotype using the following formula:


$$\frac{\text{sum}\;\left(\text{number of samples}\;\text{per}\;\text{serotype}\right)}{\text{sum}\;\left(\text{total number of samples serotyped}\right)\;}*100$$

whereby the “sum” corresponds to the total number of samples across studies included in the corresponding analysis.

For each clinical manifestation, studies were categorized into 2 vaccine periods—post-PCV7 or post-PHiD-CV/PCV13 (pooled)—based on information provided in the publications. To increase robustness and address limitations in interpreting data from settings with low PCV uptake, the primary analysis for each clinical manifestation was restricted to data from countries where the PCV was implemented through infant NIPs; studies from countries with PCV introduction limited to private markets were excluded. A sensitivity analysis was conducted for each clinical manifestation on all identified studies, including those in countries that implemented PCVs in the private market only. Furthermore, subgroup analyses on all eligible studies were conducted based on the PCV product (PHiD-CV or PCV13) for each clinical manifestation.

Serotype-specific pooled percentage averages were reported only if data from at least 5 studies were available for that specific serotype, to ensure that serotype distribution was based on a sufficiently robust number of studies and to reduce the influence of isolated or potentially biased findings. This criterion was not applied for CAP-related analyses given the limited number of studies. Similarly, it was not applied to the subgroup analyses per PCV product.

All analyses were performed with R studio and were descriptive. No formal statistical testing was performed because of the methodological heterogeneity in design, sampling methods, and population selection of the included studies. The data extraction procedure and study categorization are detailed in Supplementary methods.

## 3 Results

### 3.1 Study selection and characteristics

**Figure 1:**
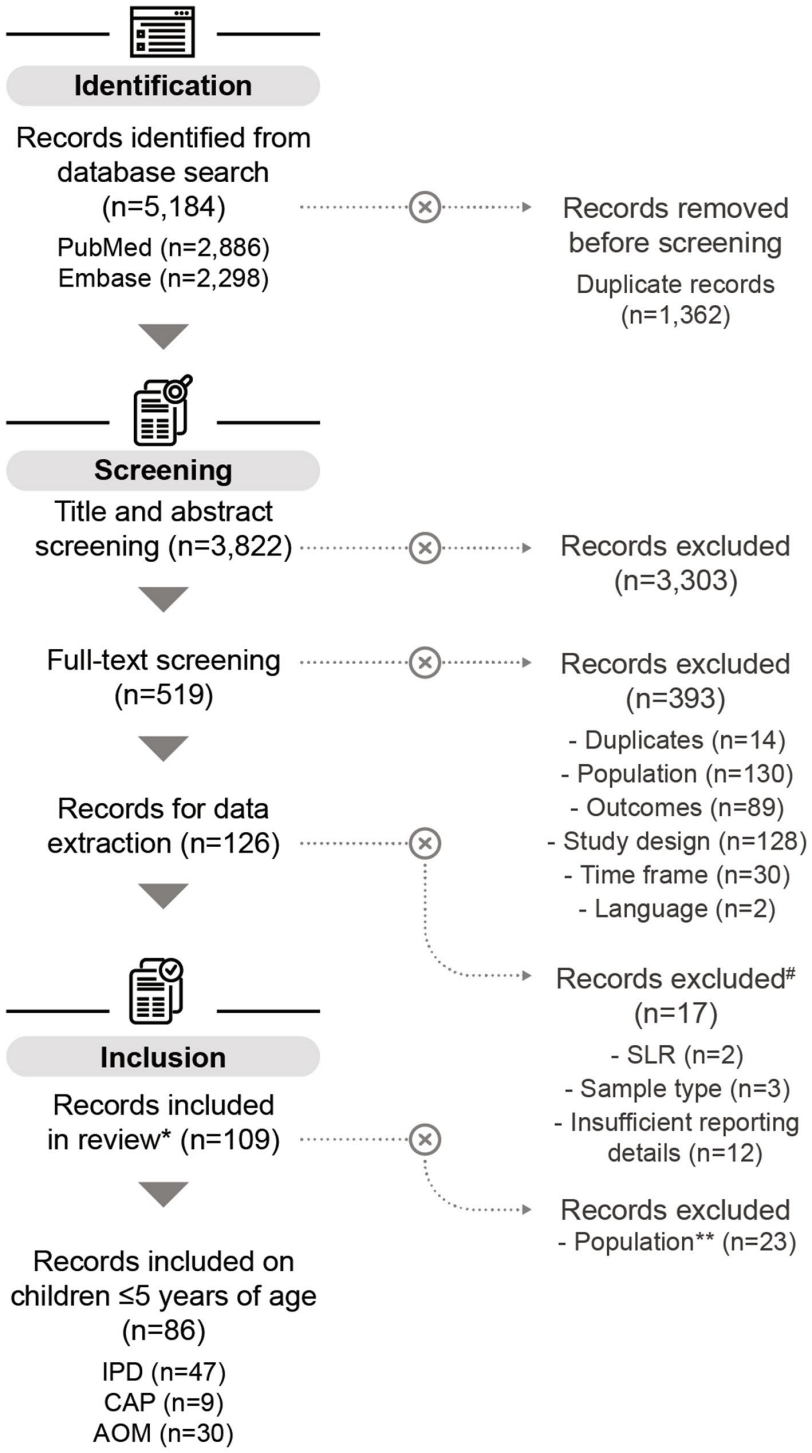
PRISMA flow chart of the systematic literature search. ^#^SLRs on IPD, records on AOM that exclusively reported serotypes from nasopharyngeal samples, and records with insufficient reporting details, were excluded from the data analysis. More information can be found in the methods section. *Some of the records included in this review also contain data on adults ≥65 years (*n* = 11), but these datapoints were not included in the analysis on children ≤5 years. **Records containing only data on adults ≥65 years were excluded for the purpose of this publication. *n*, number of records; SLR, systematic literature review; IPD, invasive pneumococcal disease; CAP, community-acquired pneumonia; AOM, acute otitis media.

After screening 3,822 publications, 126 studies were selected for data extraction, of which 109 were included in the final analysis. The main reason for study exclusion was the lack of sufficient details for further analysis. Among the selected studies, 86 covered serotype distribution data in children ≤5 years. Of these, 47 reported data on IPD (46–92), 30 on AOM (93–122), and 9 on CAP (123–131) (Figure 1). Of the 86 studies included, most were conducted in Europe (*n* = 42), followed by Asia (*n* = 21), South America (*n* = 7), North America (*n* = 6), Africa (*n* = 5), the Middle East (*n* = 4), and Australia (*n* = 1).

### 3.2 IPD

#### 3.2.1 IPD study characteristics

Among the 47 IPD-related publications, 13 reported data following PCV7 implementation and 38 following PHiD-CV or PCV13 (Supplementary Table S4); 4 reported data following both PCV7 and PHiD-CV/PCV13. Overall, serotyping data from 15,511 isolates from IPD cases were extracted from the 38 post-PHiD-CV/PCV13 studies. Most of these (32/38 studies, 84%) were conducted in a setting where the PCV was implemented through an infant NIP, and where PCV7 had been used previously. Most of the included post-PHiD-CV/PCV13 studies (32/38 studies, 84%) contained post-PCV13 data (1–9 years post-introduction), and 8 (21%) contained post-PHiD-CV data (2–6 years). Two studies were conducted in a setting where both PHiD-CV and PCV13 were implemented, each providing separable data per PCV product. In one study, data were indistinguishable due to the concurrent use of both PCV products and could thus not be used for the stratified analysis per PCV product. One post-PCV13 study presented inconsistent data that rendered it unsuitable for the stratified analysis per PCV product.

#### 3.2.2 Serotype distribution in children with IPD post-PHiD-CV/PCV13 uptake

**Figure 2:**
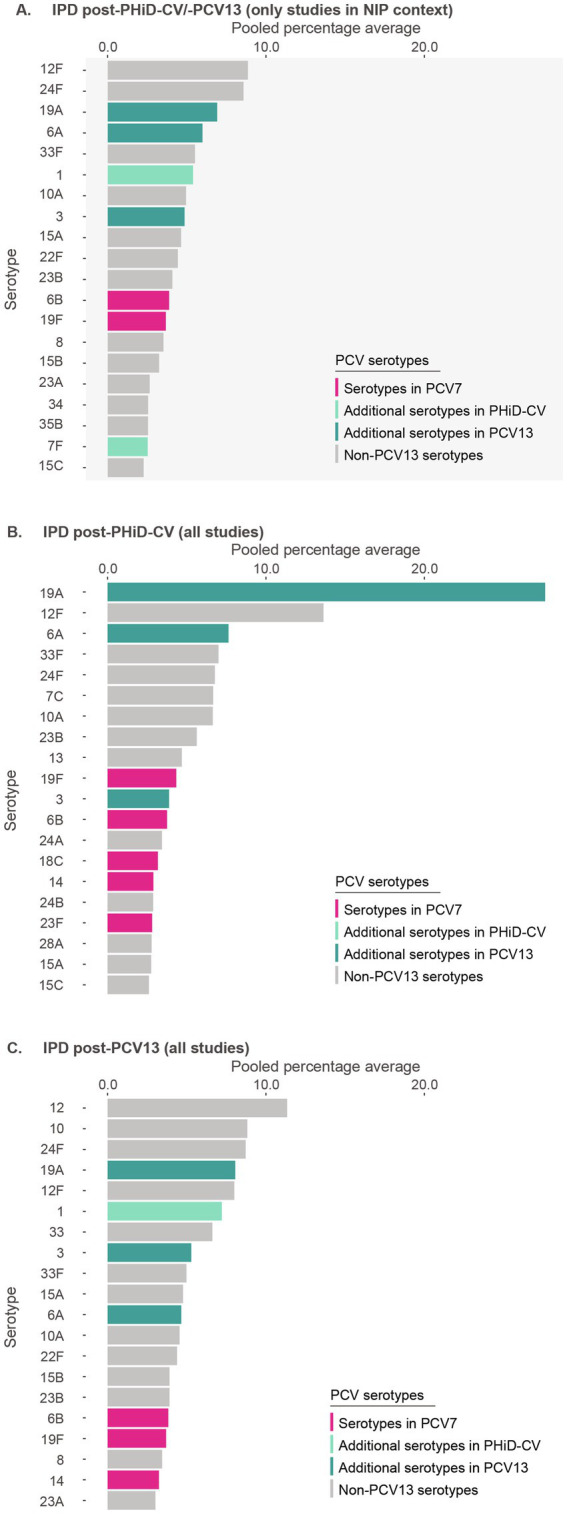
Serotype distribution in invasive pneumococcal disease among children ≤5 years of age **(A)** post-PHiD-CV/PCV13 implementation through infant national immunization programs (*n* = 32), **(B)** post-PHiD-CV (*n* = 7), and **(C)** post-PCV13 (*n* = 31) uptake in infants (either through infant national immunization programs or private markets). The top 20 serotypes are shown. Serotypes are represented by colors corresponding to the lowest valency PCV product in which they are included. In the PCV legend, the additional serotypes included in the product are relative to the next lower valency product. Pooled percentage averages were calculated for each serotype individually, thus the sum of all serotypes may exceed 100%. For panel **A**, serotype-specific pooled percentage averages were calculated only if 5 or more studies reported on the respective serotype. For panel **B** and **C**, the pooled percentage averages were calculated irrespective of the number of studies reporting on it. For panel **B**, although the legend includes all PCVs, no PHiD-CV-specific serotypes were identified in the top-20 serotypes. IPD, invasive pneumococcal disease; *n*, number of studies that were included in the analysis; NIP, national immunization program; PCV, pneumococcal conjugate vaccine; PCV7, 7-valent PCV; PCV13, 13-valent PCV; PHiD-CV, pneumococcal non-typeable *Haemophilus influenzae* protein D conjugate vaccine.

Post-PHiD-CV/PCV13 implementation through infant NIPs (primary analysis, 32 studies), the top 10 serotypes responsible for causing IPD were 12F (pooled percentage average of 8.9%), 24F (8.6%), 19A (6.9%), 6A (6.0%), 33F (5.5%), 1 (5.4%), 10A (5.0%), 3 (4.9%), 15A (4.6%), and 22F (4.4%) (Figure 2A, Supplementary Table S5). The sensitivity analysis, including 6 additional studies where the PCV was introduced to the private market only (38 studies), showed a shift in the rankings of these serotypes. In this analysis, serotype 19A (9.9%) was the leading serotype, followed by 24F (8.4%), 12F (8.3%), 1 (6.5%), 33F (5.2%), 3 (5.1%), 6A (4.9%), 10A (4.7%), 15A (4.5%), and 22F (4.2%) (Supplementary Figure S1). Overall, this suggests that vaccine implementation through NIPs, and consequently increasing vaccine uptake, provided additional impact on vaccine-type disease, particularly on serotype 19A. Nevertheless, post-PHiD-CV/PCV13, serotypes 19A, 6A, and 3, included in PCV13, and serotype 1, included in both PHiD-CV and PCV13, still contributed frequently to remaining IPD (Figure 2A, Supplementary Figure S1).

In PHiD-CV settings, serotype 19A was prominently reported (27.6%), followed by serotypes 6A (7.6%), 3 (3.9%), and 1 (1.0%) (Figure 2B). In PCV13 settings, serotype 19A still contributed to disease, but at much lower frequency (8.1%) (Figure 2C), with differing contributions for serotypes 6A (4.7%), 3 (5.3%), and 1 (7.3%).

Post-PCV7 in NIP settings, 19A was identified as the leading serotype (24.7%), followed by serotype 24F (15.0%) (Supplementary Figure S2).

### 3.3 AOM

#### 3.3.1 AOM study characteristics

Among the 30 included AOM-related publications, 22 reported serotype distribution data following the adoption of PCV7 and 8 following PHiD-CV or PCV13 (Supplementary Table S4). Two studies reported data from both PCV periods. Overall, data from 731 serotyped isolates from AOM cases were extracted from the 8 post-PHiD-CV/PCV13 studies, which were all conducted in NIP setting. Of these, 5 studies (63%) were in a setting of PCV13 use (4–8 years post-introduction), 2 (25%) in a setting of PHiD-CV use (6–7 years), and 1 in context of mixed PHiD-CV/PCV13 use (4 years). The post-PHiD-CV studies were conducted in locations without previous PCV7 use, while the PCV13 studies were in a location with previous PCV7 use.

#### 3.3.2 Serotype distribution in children with AOM post-PHiD-CV/PCV13 uptake

**Figure 3:**
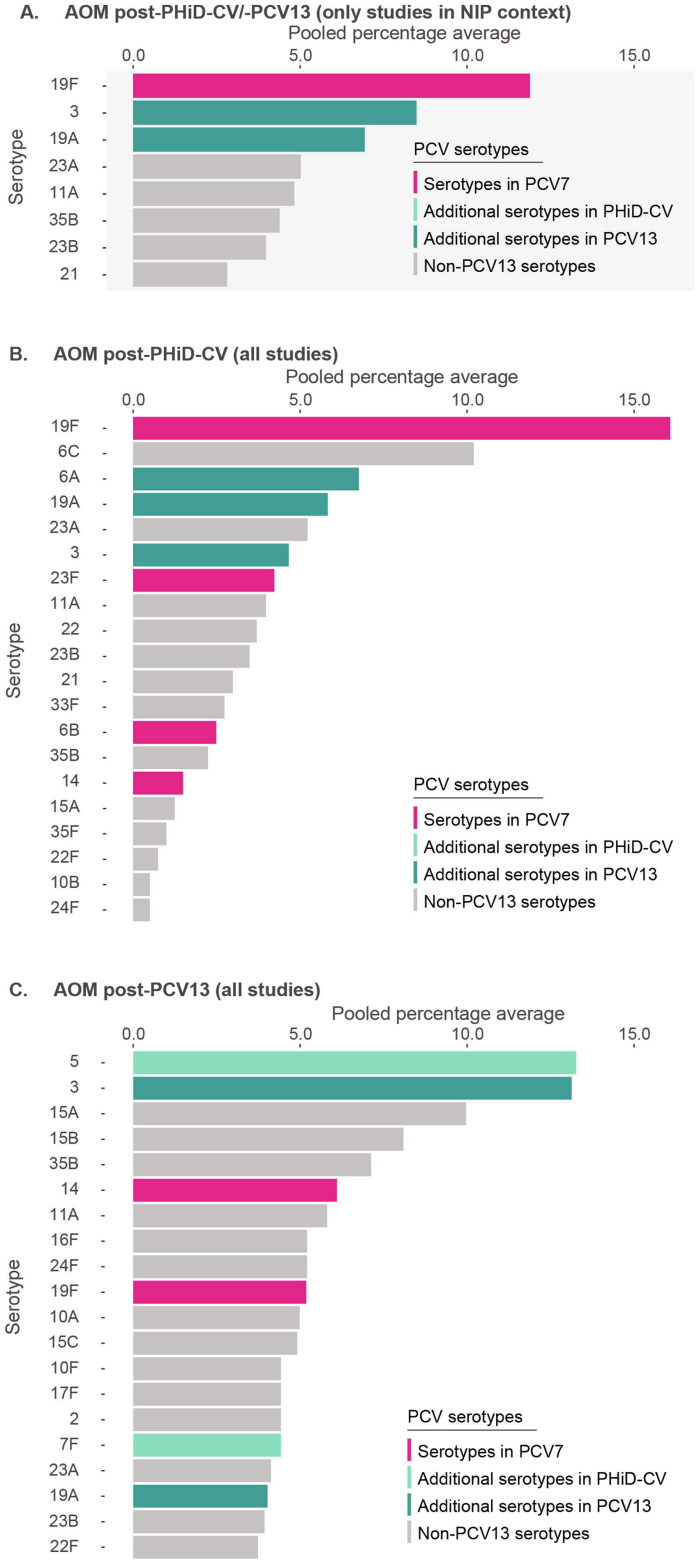
Serotype distribution in acute otitis media among children ≤5 years of age **(A)** post-PHiD-CV/PCV13 implementation through infant national immunization programs (*n* = 8), **(B)** post-PHiD-CV (*n* = 2), and **(C)** post-PCV13 (*n* = 5) uptake in infants (either through infant national immunization programs or private markets). The top 20 serotypes are shown. Serotypes are represented by colors corresponding to the lowest valency PCV product in which they are included. In the PCV legend, the additional serotypes included in the product are relative to the next lower valency product. Pooled percentage averages were calculated for each serotype individually, thus the sum of all serotypes may exceed 100%. For panel **A**, serotype-specific pooled percentage averages were calculated only if 5 or more studies reported on the respective serotype. For panel **B** and **C**, the pooled percentage averages were calculated irrespective of the number of studies reporting on it. AOM, acute otitis media; *n*, number of studies that were included in the analysis; NIP, national immunization program; PCV, pneumococcal conjugate vaccine; PCV7, 7-valent PCV; PCV13, 13-valent PCV; PHiD-CV, pneumococcal non-typeable *Haemophilus influenzae* protein D conjugate vaccine.

Post-PHiD-CV/PCV13 implementation, 8 serotypes were reported: 19F (11.9%), 3 (8.5%), 19A (6.9%), 23A (5.0%), 11A (4.8%), 35B (4.4%), 23B (4.0%), and 21 (2.8%) (Figure 3A, Supplementary Table S6). This suggests that serotype 19F, included in both PHiD-CV and PCV13, as well as serotypes 3 and 19A, included in PCV13, contributed frequently to remaining AOM.

In PHiD-CV settings, the contribution of serotypes 19F, 3, and 19A was 16.1, 4.7, and 5.8%, respectively (Figure 3B). In PCV13 settings, their contribution was 5.9, 14.8, and 4.5%, respectively (Figure 3C).

Post-PCV7, serotype 19A clearly dominated rankings (30.5%), followed by serotypes 3 (9.2%) and 19F (7.7%) (Supplementary Figure S3).

### 3.4 CAP

#### 3.4.1 CAP study characteristics

Nine CAP-related studies were identified, of which 6 were performed following the adoption of PCV7 and 3 following PHiD-CV or PCV13 (Supplementary Table S4). Most CAP studies identified Spn from blood cultures, confirming bacteremic CAP cases. Overall, data from 235 serotyped isolates from CAP cases were extracted post-PHiD-CV/PCV13, all from studies in NIP context. Of the 3 post-PHiD-CV/PCV13 studies, 1 (33%) contained data in PHiD-CV setting (5 years of use), and 2 (67%) in PCV13 setting (6–7 years). The post-PHiD-CV study was conducted in a location without previous PCV7 use; the PCV13 studies were conducted in a location with previous PCV7 use.

#### 3.4.2 Serotype distribution in children with CAP post-PHiD-CV/PCV13 uptake

**Figure 4:**
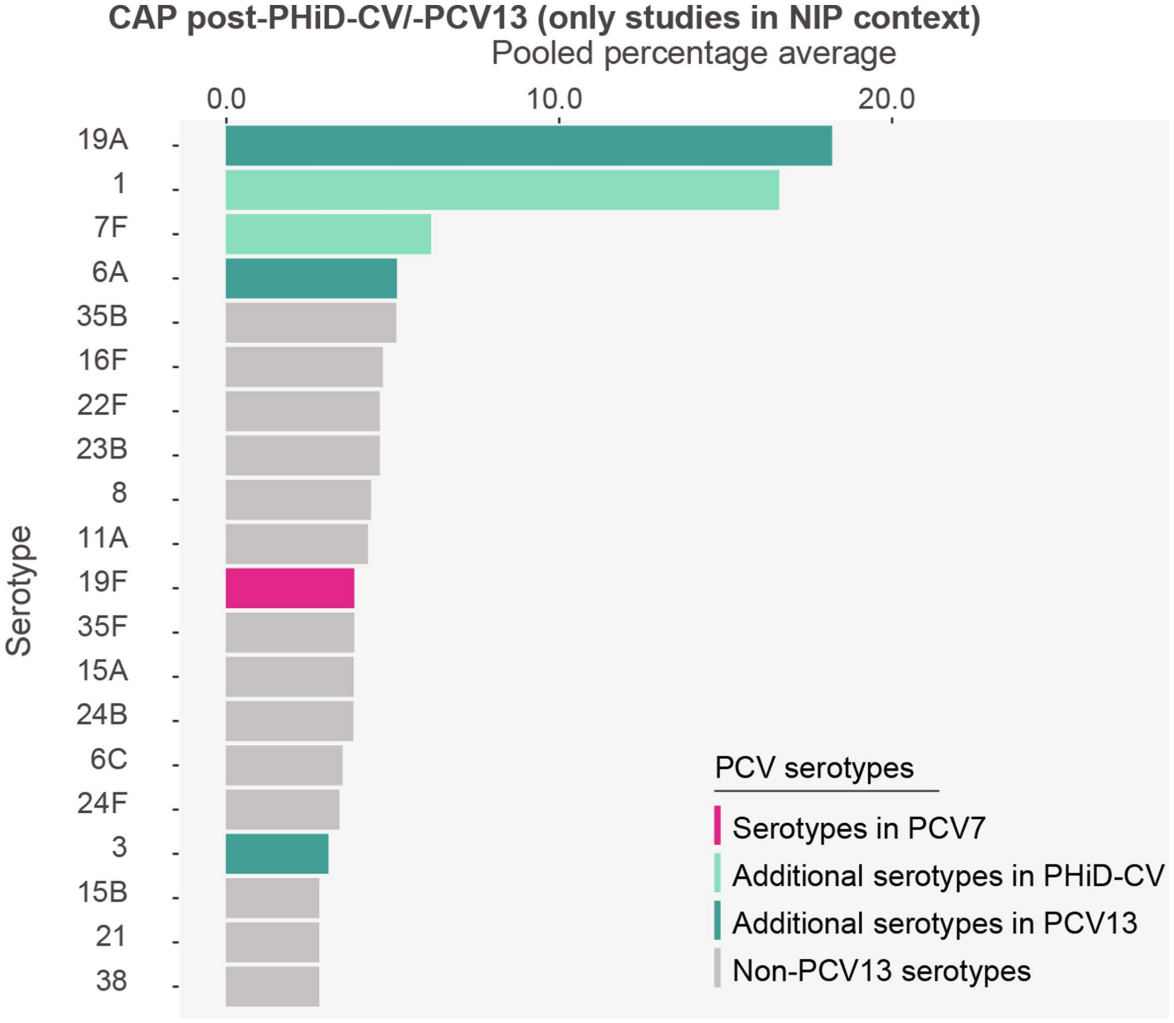
Serotype distribution in community-acquired pneumonia among children ≤5 years of age post-PHiD-CV/-PCV13 implementation through infant national immunization programs (*n* = 3). The top 20 serotypes are shown. Serotypes are represented by colors corresponding to the lowest valency PCV product in which they are included. In the PCV legend, the additional serotypes included in the product are relative to the next lower valency product. Pooled percentage averages were calculated for each serotype individually, thus the sum of all serotypes may exceed 100%. Serotype-specific pooled percentage averages were calculated irrespective of the number of studies reporting on it. Given the limited number of studies on CAP, results from the subgroup analyses per PCV product need to be interpreted with caution and are included in the Supplementary results. CAP, community-acquired pneumonia; n, number of studies that were included in the analysis; PCV, pneumococcal conjugate vaccine; PCV7, 7-valent PCV; PHiD-CV, pneumococcal non-typeable *Haemophilus influenzae* protein D conjugate vaccine; PCV13, 13-valent PCV.

Post-PHiD-CV/PCV13 adoption, serotypes 19A (18.2%) and 1 (16.6%) were the most frequently reported serotypes in CAP cases. These were followed by serotypes 7F (6.2%), 6A (5.1%), 35B (5.1%), 16F (4.7%), 22F (4.6%), 23B (4.6%), 8 (4.3%), and 11A (4.3%) (Figure 4, Supplementary Table S7).

Serotype 19A was the main serotype in the PHiD-CV setting (28.2%) (Supplementary Figure S4), while serotype 1 was predominant in PCV13 settings (24.2%) (Supplementary Figure S5).

Post-PCV7, serotypes 1 (21.0%) and 19A (19.7%) were predominantly identified (Supplementary Figure S6).

## 4 Discussion

Our SLR indicates that despite widespread PCV implementation, some vaccine serotypes were still frequently reported. Particularly serotype 19A, which dominated rankings across all clinical manifestations post-PCV7, was still commonly identified, although its pooled percentage average appeared reduced in PCV13 settings. Serotype 3 continued to be regularly detected in IPD and AOM cases post-PHiD-CV/PCV13, across both PHiD-CV and PCV13 settings. Serotype 1 was another highly common vaccine serotype detected in IPD and CAP cases in the post-PCV7 era, that persisted post-PHiD-CV/PCV13 adoption (mostly in PCV13 settings); in contrast, this serotype contributed minimally to AOM in either of the 2 PCV periods. In AOM, serotype 19F, which was already included in PCV7, remained prevalent post-PHiD-CV/PCV13 implementation, though mostly in PHiD-CV settings without previous PCV7 use.

Our study also indicated an increase in several non-PCV13 serotypes post-PHiD-CV/PCV13 introduction compared to the post-PCV7 era. In IPD, serotypes 12F and 24F were frequently reported. Common non-PCV13 serotypes detected in AOM and CAP were 11A, 35B, and 23B.

This SLR presents a comprehensive summary that may extend beyond the pivotal PCV Review of Impact Evidence (PRIME) SLR on IPD conducted by the WHO in 2017 (132). Our review covers a broader scope of serotype-specific data across the 3 major clinical manifestations of pneumococcal disease—namely IPD, AOM, and CAP. While the PRIME report, based on published impact and effectiveness data up to 2017, underscored the impact of PCV13 on serotype 19-mediated IPD, it noted insufficient evidence to evaluate the impact on serotypes 3 and 6A. By extending our analyses up to 2020, we potentially captured more recent insights on these serotypes, and on those not covered in the earlier PRIME analysis.

Parallels of our IPD data were also noted with the ongoing, global Pneumococcal Serotype Replacement and Distribution Estimation (PSERENADE) project, (22, 133) a significant IPD study that used “raw” surveillance data potentially less influenced by serotype reporting bias (22, 133). Similar to our observations, these recent data collected between 2015 and 2018 show that serotypes 19A and 3 were the leading serotypes, with serotype 19A mainly detected in PHiD-CV settings and serotype 3 in both PHiD-CV and PCV13 settings. Serotype 6C was among the leading serotypes at PHiD-CV sites in the PSERENADE study, but scarcely reported in both PHiD-CV and PCV13 settings in our IPD analysis. In contrast to our analysis, serotypes 6A and 1 were contributing minimally to IPD in the PSERENADE study, which may be due to differences in geographical diversity or maturity of the PCV programs. Our IPD findings also align with other SLRs/meta-analyses that showed that serotypes 19A and 3 were the main IPD serotypes post-PHiD-CV/PCV13 (41, 134–137). Furthermore, several of the most prominent non-PCV13 serotypes reported by the PSERENADE project (22, 133) and another SLR on individual serotypes (41)—in particular 10A, 12F, 22F, 24F, and 33F—were also highly ranked in our IPD analysis.

AOM and CAP represent the largest burden of pneumococcal disease, but serotype distribution data in these manifestations are scarce. Our AOM analysis confirmed the observation of a recent AOM-focused SLR that 19F, 3, and 19A were the predominant PCV13 serotypes associated with AOM post-PHiD-CV/PCV13 implementation (138).

While our CAP analysis should be interpreted with caution due to the low number of included studies, PCV13 serotypes 1 and 19A still seemed to dominate rankings in the post-PHiD-CV/PCV13 period, similar to the post-PCV7 period. Serotype 3, commonly reported post-PCV7 in CAP cases, was nearly not reported post-PHiD-CV/PCV13. Given that our search strategy for CAP studies mostly retrieved publications on invasive cases, our CAP data might be largely representative of this smaller CAP population (139). Importantly, the CAP and IPD results might be partially overlapping, since 12–16% of IPD patients aged <2 years are estimated to have invasive CAP (140).

The persistence of disease caused by particular vaccine serotypes is probably multifactorial. One factor may be the variation in PCV effectiveness for different serotypes, e.g., PCV13 effectiveness against serotype 3 has not been consistently demonstrated (134, 141) and PHiD-CV has not been effective at controlling 19A-mediated disease (11, 142). Although our analysis excluded studies involving immunocompromised children and those with other comorbidities, which are well-known risk factors for vaccine failure (143–145), the remaining vaccine-type disease may be partially attributed to unreported underlying comorbidities or other individual risk factors (146–149). Lastly, studies have suggested that vaccine and antimicrobial pressure can both induce clonal changes and capsular switching, leading to the genetic transformation of virulent vaccine serotypes into variants that escape vaccine-mediated immunity, thereby being able to occupy the ecological niche (150–156).

PCV15 and PCV20 were recently approved for use in children and adults in different countries. Also the first pneumococcal vaccine specifically designed for adults, a 21-valent PCV (PCV21, *Capvaxive*, Merck Sharp & Dohme LLC, a subsidiary of Merck & Co, Inc., [MSD]), has been recently introduced (157). In addition to these, some regionally used PCVs are contributing to the expanding pneumococcal vaccine landscape, though their broader impact remains more limited (158, 159). Considering that the full impact of a PCV is only evident after about 4 years, provided high immunization rates are achieved, it is too soon to evaluate changes in pneumococcal epidemiology following the introduction of these new PCVs (19). In addition, since PCV15 and PCV20 were licensed based on immunological non-inferiority compared to PCV13, their potential benefit on disease remains to be determined (157, 160–164). Current pneumococcal vaccine technologies have also been shown to exert carrier-induced immune suppression—a phenomenon in which the antibody response to the carrier protein compromises the response to the serotype polysaccharide—which increases as the number of glycoconjugates (valencies) included in PCVs increases (165). This may explain why phase 3 trials evaluating 3- and 4-dose infant vaccination series of PCV20 showed that immune responses to some serotypes did not meet some pre-specified statistical non-inferiority criteria compared to PCV13 (162, 163). Our results also highlight differences in pneumococcal epidemiology across the different clinical manifestations. Current PCVs do not cover the full spectrum of pneumococcal diseases, potentially leaving gaps in protection. Additional factors amplifying serotype diversity, such as geographical location and the age of the at-risk population (41, 135), make it challenging for current vaccines to provide complete protection. Therefore, novel pneumococcal vaccination strategies and technologies are needed that can provide enhanced and broader protection.

Our review has several limitations. As the search on studies was limited to those published up to 2020, the collected data might not reflect the most up-to-date pneumococcal epidemiology. Nevertheless, this time restriction was chosen to avoid introducing effects of the COVID-19 pandemic, as the social preventive measures that were applied led to an intermittent global decrease in Spn transmission and subsequent rebound effect (16). Several studies evaluating serotype distribution in IPD cases after relaxation of the preventive measures indicated a generally similar serotype distribution as before the pandemic. IPD was mainly caused by non-PCV13 serotypes, but several vaccine serotypes (including 3 and 19A) were still prominent (166–170).

It was not feasible to accurately account for the heterogeneity inherent to observational studies. Overall, the study heterogeneity did not allow us to perform a meta-analysis. Instead, serotype-specific pooled percentage averages were calculated for this descriptive analysis, which considered for each serotype the sizes of the studies reporting on the respective serotype. This approach accounts for the varying availability of data for each serotype across studies, which allows for an estimation that reflects the prevalence of each serotype within the subset of studies reporting on it. However, it is a less robust approach compared to meta-analysis, and it does not allow for confirmatory statistical analyses or conclusive outcomes. Additionally, it may disproportionally bias the serotype distribution to certain serotypes with higher reporting rates. For our main post-PHiD-CV/PCV13 analyses, we focused only on serotypes that were reported by at least 5 studies, to reduce the impact of isolated or potentially biased findings. Nevertheless, this may have resulted in the omission of an important emerging serotype reported in a small number of studies. In addition, our analyses are largely driven by studies in high-income countries, which mostly have high-quality surveillance systems in place. As serotype circulation, vaccination programs and uptake, and pneumococcal disease burden differ in developing countries, this may limit the generalizability of our findings (21, 171). Lastly, the availability of studies conducted in PHiD-CV settings was limited and unbalanced in comparison to those conducted in PCV13 settings. Therefore, our analyses remained descriptive, and the outcomes and comparisons should be interpreted with caution.

In conclusion, while the overall incidence of pneumococcal disease has consistently declined with the introduction of PHiD-CV and PCV13, the serotype distribution responsible for remaining disease has changed, with non-PCV13 serotypes becoming predominant. Several vaccine serotypes—in particular serotypes 19A, 1, and 3—are still responsible for a substantial proportion of remaining invasive and non-invasive pneumococcal disease. Continued monitoring of serotype evolution therefore remains critical to appraise optimal vaccination strategies for the prevention of pneumococcal disease, including new vaccine technologies that could provide broader and improved protection in children.

## Trademark statement

*Synflorix* is a trademark licensed to or owned by GSK. *Prevenar/Prevnar*, *Prevenar 13/Prevnar 13* and *Prevenar 20/Prevnar 20* are trademarks of Pfizer Inc. *Vaxneuvance* and *Capvaxive* are trademarks of Merck Sharp & Dohme LLC, a subsidiary of Merck & Co., Inc. (MSD).
