# Supplementary material for: Serotype distribution of invasive and non-invasive pneumococcal disease in children ≤5 years of age following the introduction of 10- and 13-valent pneumococcal conjugate vaccines in infant national immunization programs: a systematic literature review
Source: Front Public Health. 2025 May 30;13:1544359. doi: 10.3389/fpubh.2025.1544359 (PMC12162640; doi:10.3389/fpubh.2025.1544359)
Supplement: Supplementary file 1 [file Data_Sheet_1.docx]

Supplementary Material

# Supplementary methods

## Detailed search strategies

PubMed search strategy

1. (serotype[Title/Abstract]) AND (((PCV[Title/Abstract]) OR (PHiD-CV[Title/Abstract]) OR (10-valent[Title/Abstract]) OR (PHID-CV[Title/Abstract]) OR (13-valent[Title/Abstract]) OR (PCV13[Title/Abstract])) OR ((pneumonia[Title/Abstract]) OR (IPD[Title/Abstract]) OR (Otitis[Title/Abstract]) OR (invasive pneumococcal disease[Title/Abstract])))

2. ((etiology[Title/Abstract]) AND (Streptococcus pneumoniae[Title/Abstract])) AND (((PCV[Title/Abstract]) OR (PHiD-CV[Title/Abstract]) OR (10-valent[Title/Abstract]) OR (PHID-CV[Title/Abstract]) OR (13-valent[Title/Abstract]) OR (PCV13[Title/Abstract])) OR ((pneumonia[Title/Abstract]) OR (IPD[Title/Abstract]) OR (Otitis[Title/Abstract]) OR (invasive pneumococcal disease[Title/Abstract])))

Embase search strategy


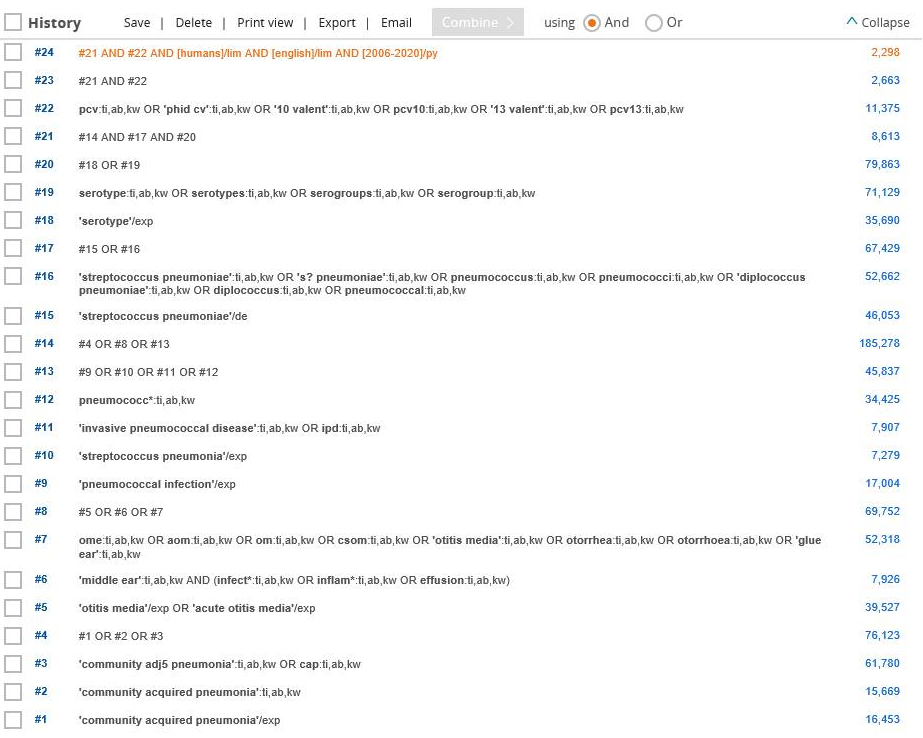


## List of inclusion and exclusion criteria

**Table S1.** IPD eligibility criteria

|  | **Inclusion** | **Exclusion** |
| --- | --- | --- |
| **Population** | - IPD^#^ in children ≤5 years   - The study population (hospitalized or non-hospitalized) should be representative of the general population, not a specific group with comorbidities   - Studies in children <18 years can be included if the median age is 2 years or there is an indication that the majority of the children are ≤5 years   - Method for diagnosing IPD must be clearly stated for a study to be included | - Immunocompromised individuals - Other infectious disease - Sub-population with comorbidities - Studies that do not report the Spn distribution separately for IPD or segregate the distribution for otherwise healthy individuals from immunocompromised individuals - Studies on other age groups and/or that do not report serotype distribution for the age group of interest - Studies that do not state or clearly state the diagnostic method for IPD |
| **Outcome** | - Spn serotypes post-PCV implementation | - Spn serotypes pre-PCV implementation - Other pathogens - Antibiotic resistant Spn strains |
| **Study design** | - SLRs - Meta-analyses - Observational studies with**:** - At least 30 serotyped isolates - Serotyping on samples obtained from sterile sites, e.g., blood, cerebrospinal fluid, pleural fluid, joint fluid, pericardial fluid, etc. | - Other study designs, including but not limited to RCTs, narrative reviews, opinions, editorials, case reports, carriage studies, genomic studies, animal studies, assay development studies - Studies reporting on <30 serotyped isolates or that do not report the total number of serotyped isolates - Studies that do not report different serotypes or groups for IPD - Quarterly or province-level surveillance report, if annual and/or national report were available - Surveillance reports published on surveillance websites - Studies with lack of detailed description about the serotypes of the isolates - Serotyping on samples obtained from non-sterile sites, e.g., nasopharynx or oropharynx, sputum, urine, etc. |
| **Time frame** | - 1 January 2006 to 31 December 2020 (SLRs and meta-analyses), or - 1 January 2018 to 31 December 2020 (observational studies) | - Pre 2006 or post 2020 |
| **Language** | - English language | - Non-English language |
| **Region** | - Global | - NA |

^#^ IPD was defined as the isolation of Spn from sterile sites such as blood, cerebrospinal fluid, pleural fluid, joint fluid, or pericardial fluid (1).

IPD, invasive pneumococcal disease; NA, not applicable; PCR, polymerase chain reaction; PCV, pneumococcal conjugate vaccine; RCT, randomized controlled trials; SLR, systematic literature review; Spn, *Streptococcus pneumoniae*

**Table S2.** AOM eligibility criteria

|  | **Inclusion** | **Exclusion** |
| --- | --- | --- |
| **Population** | - AOM^#^ in children ≤5 years   - The study population (hospitalized or non-hospitalized) should be representative of the general population, not a specific group with comorbidities   - Studies on children <18 years, can be included if the median age is 2 years or there is an indication that the majority of the children are ≤5 years   - Method for diagnosing AOM must be clearly stated for a study to be included | - Immunocompromised individuals - Other infectious disease - Sub-population with comorbidities - Studies that do not report the Spn distribution separately for AOM or segregate the distribution for otherwise healthy individuals from immunocompromised individuals - Studies on other age groups and/or that do not report serotypes distribution for the age group of interest - Studies that do not state or clearly state the diagnostic method for AOM |
| **Outcome** | - Spn serotypes post-PCV implementation | - Spn serotypes pre-PCV implementation - Other pathogens - Antibiotic resistant Spn strains |
| **Study design** | - Observational studies with:   - At least 30 serotyped isolates   - Serotyping on samples obtained from middle ear fluid, originating from tympanocentesis/tympanostomy, or spontaneous perforation | - Other study designs, including but not limited to RCTs, SLRs, meta-analyses, narrative reviews, opinions, editorials, case reports, carriage studies, genomic studies, animal studies, assay development studies - Studies reporting on <30 serotyped isolates or that do not report the total number of serotyped isolates - Serotyping on samples obtained from other sites or sources, e.g., blood, cerebrospinal fluid, urine, nasopharynx etc. - Studies that do not report different serotypes or groups for AOM - Quarterly or province-level surveillance report, if annual and/or national report were available - Surveillance reports published in surveillance websites - Studies with lack of detailed description about the serotypes of the isolates |
| **Time frame** | - 1 January 2006 to 31 December 2020 | - Pre 2006 or post 2020 |
| **Language** | - English language | - Non-English language |
| **Region** | - Global | - NA |

^#^ AOM was defined as the presence of inflammation of the middle ear, associated with an effusion, accompanied by rapid onset of symptoms and signs of an ear infection (2, 3).

AOM, acute otitis media; NA, not applicable; PCR, polymerase chain reaction; PCV, pneumococcal conjugate vaccine; RCT, randomized controlled trial; SLR, systematic literature review; Spn, *Streptococcus pneumoniae.*

**Table S3**. CAP eligibility criteria

|  | **Inclusion** | **Exclusion** |
| --- | --- | --- |
| **Population** | - CAP^#^ in children ≤5 years   - The study population (hospitalized or non-hospitalized) should be representative of the general population, not a specific group with comorbidities   - Studies on children <18 years, can be included if the median age is 2 years or there is an indication that the majority of the children are ≤5 years   - Method for diagnosing CAP must be clearly stated for a study to be included | - Immunocompromised individuals - Other infectious disease - Sub-population with comorbidities - Studies that do not report the Spn distribution separately for CAP or segregate the distribution for otherwise healthy individuals from immunocompromised individuals - Studies on other age groups and/or that do not report serotypes distribution for the age group of interest - Studies that do not state or clearly state the diagnostic method for CAP |
| **Outcome** | - Spn serotypes post-PCV implementation | - Spn serotypes pre-PCV implementation - Other pathogens - Antibiotic resistant Spn strains |
| **Study design** | - Observational studies with:   - At least 20 serotyped isolates   - Serotyping on samples obtained from sterile and non-sterile sites     - Sterile sites: blood, cerebrospinal fluid, pleural fluid, joint fluid, pericardial fluid, etc.     - Non-sterile sites: sputum, bronchial aspirates, biopsy samples, etc. | - Other study designs, including but not limited to RCTs, SLRs, meta-analyses, narrative reviews, opinions, editorials, case reports, carriage studies, genomic studies, animal studies, assay development studies - Studies reporting on <20 serotyped isolates or that do not report the total number of serotyped isolates - Studies that do not report different serotypes or groups for CAP - Quarterly or province-level surveillance report, if annual and/or national report were available - Surveillance reports published in surveillance websites - Studies with lack of detailed description about the serotypes of the isolates - Serotyping on samples obtained from other sites or sources |
| **Time frame** | - 1 January 2006 to 31 December 2020 | - Pre 2006 or post 2020 |
| **Language** | - English language | - Non-English language |
| **Region** | - Global | - NA |

^#^ CAP was defined as pneumonia acquired outside of the hospital (4).

CAP, community-acquired pneumonia; NA, not applicable; PCR, polymerase chain reaction; PCV, pneumococcal conjugate vaccine; RCT, randomized controlled trial; SLR, systematic literature review; Spn, *Streptococcus pneumoniae*

## Detailed study selection workflow

After removal of duplicates, the titles and abstracts of the obtained publications were independently screened by two reviewers against the eligibility criteria. Any discrepancies between the reviewers were discussed, and if not resolved, a third reviewer made the final decision. The full texts of all selected publications were then screened by a single reviewer using the same eligibility criteria.

For the purpose of this paper, studies including data on children aged ≤5 years were retained. If publications with overlapping data were identified, the publication with the highest number of pneumococcal isolates and/or longest study period was selected.

## Detailed data extraction workflow

A single reviewer extracted the relevant data from each publication using DistillerSR (5), which included: publication details, country or region of data collection, data collection period, study design, study setting, clinical manifestation (i.e., IPD, CAP, or AOM), certainty of diagnosis (i.e., laboratory confirmed, clinical diagnosis only, mentioning of ‘suspected’ Spn), age of the study population, patient sample size (if available per age), serotype detection method, clinical specimen type, total number of isolates serotyped, number of isolates per serotype, number of Spn serotypes detected, PCV product used in the country at the time of the study (i.e., PCV7, PHiD-CV, or PCV13), previous PCV7 implementation, duration from initiation of PHiD-CV/PCV13 implementation via NIPs in the country until study end, study limitations, and study conclusion. Relevant findings were reported using the terminology, numbers, and percentages included in the publications. When the number and/or percentage of the pneumococcal serotypes were only reported in a graphical format, the data were extracted using WebPlot Digitizer (6).

A second independent reviewer performed a quality control check on all the extracted data. The risk of bias for the included studies was not systematically assessed. The extracted data was exported from DistillerSR into a spreadsheet.

## Categorization of studies for data analysis

For categorizing studies in vaccine periods (i.e., post-PCV7 and post-PHiD-CV/-PCV13), information provided in the publications were used. When data were reported on both PCV periods without a breakdown of serotypes specific to each PCV period, the PCV period that covered the majority of the study duration was selected. “Transitional” periods (i.e., a period in which a country was substituting PCV7 for PHiD-CV or PCV13) were omitted from the analysis, as this period could involve a mixed schedule of PCV7 with PHiD-CV or PCV13 for some children.

The selection of studies for primary analyses, which were limited to those conducted within NIP frameworks, was guided by information from both the VIEW-hub vaccine information platform from the International Vaccine Access Center (IVAC) (7) and details provided within each publication. When studies reported breakdowns across different phases of PCV implementation (i.e., licensed use, targeted use in high-risk groups only, early routine use, late routine use), only data from the early and late PCV periods, as defined in the respective publication, were included in the primary analysis.

# Supplementary results

**Table S4.** Characteristics of the included post-PHiD-CV/PCV13 studies

| Author, year (ref) | Study design | Country | Median age | Age range | Sample type | N | Data collection period | Previous PCV7 use | PHiD-CV /PCV13 | NIP* | Years of PHiD-CV/ PCV13 use via NIP |
| --- | --- | --- | --- | --- | --- | --- | --- | --- | --- | --- | --- |
| *IPD* |  |  |  |  |  |  |  |  |  | ***84%*** |  |
| *n=38* |  |  |  |  |  | ***15,511*** |  | ***n=32*** |  | ***n=32*** |  |
| Amin-Chowdhury et al. 2020 (8) ^1^ | Prospective cohort | UK | NR | <5Y^1^ | Blood/CSF/pleural fluid | 994 | 2014/2015–2017/2018 | Yes | PCV13 | Yes | 8 |
| Baxter et al. 2021 (9) | Surveillance | USA | NR | ≥6W–<6Y | Blood/CSF/joint fluid/pericardial fluid/pleural fluid/peritoneal fluid/surgical aspirate/bone | 118 | 2010–2018 | Yes | PCV13 | Yes | 8 |
| Berezin et al. 2020 (10) | Retrospective cohort | Brazil | NR | 0–15Y | Blood/CSF/joint fluid/pleural fluid/ascitic fluid | 62 | 2011–2015 | Yes | PHiD-CV | Yes | 5 |
| Ceyhan et al. 2020 (11) ^2^ | Cross-sectional | Turkey | NR | <18Y | Blood/CSF/pleural fluid | 270 | 2015–2018 | Yes | PCV13 | Yes | 7 |
| Cohen et al. 2021 (12) | Prospective cohort and surveillance | France | 29.6M | 6M–10Y | Sterile site | 1,385 | 2012–2018 | Yes | PCV13 | Yes | 8 |
| Corcoran et al. 2020 (13) | Surveillance | Ireland | 37.7M | ≤16Y | Sterile site | 380 | 2007–2018 | Yes | PCV13 | Yes | 8 |
| de Miguel et al. 2020 (14) | Surveillance | Spain | NR | NR | NR | 189 | 2009–2019 | Yes | PCV13 | Yes | 9 |
| Desmet et al. 2021 (15) | Surveillance | Belgium | NR | <16Y | Blood | 919 | 2013–2018 | Yes | PHiD-CV/ PCV13 | Yes | 3/3 |
| Desmet et al. 2020 (16) | Surveillance | Belgium | NR | <16Y | Blood/CSF/joint fluid/pleural fluid | 365 | 2015–2018 | Yes | PHiD-CV | Yes | 3 |
| Hernandez et al. 2019 (17) | Prospective cohort | Spain | NR | 0–18Y | Sterile site | 222 | 2012–2015 | Yes | PCV13 | No | NA |
| Hernstadt et al. 2020 (18) ^3^ | Retrospective cohort | Australia | 2.2Y | 0.1–15.2Y | Blood/CSF/pleural fluid/deep abscess/ peritoneal and synovial fluid | 131 | January 1, 2011–May 7, 2017 | Yes | PCV13 | Yes | 7 |
| Iwata et al. 2020 (19) | Surveillance | Japan | NR | <18Y | CSF | 102 | 2014–2016 | Yes | PCV13 | Yes | 3 |
| Izquierdo et al. 2020 (20) | Prospective cohort | Spain | NR | 0–≤17Y | Blood/CSF/pleural fluid/articular fluid | 185 | January 2012–June 2016 | Yes | PCV13 | Yes | 6 |
| Kambiré et al. 2018 (21) | Surveillance | Burkina Faso | NR | NR | CSF | 212 | 2014–2015 | No | PCV13 | Yes | 2 |
| Kaplan et al. 2019 (22) | Surveillance | USA | NR | NR | Blood/CSF/joint fluid/pleural fluid/peritoneal fluid | 482 | 2014–2017 | Yes | PCV13 | Yes | 7 |
| Ladhani et al. 2018 (23) ^4^ | Prospective cohort | UK | NR | <5Y^2^ | Sterile site | 331 | 2016–2017 | Yes | PCV13 | Yes | 7 |
| Levy et al. 2020 (24) | Prospective cohort | France | 38.3M (2011); 20.4M (2012); 28.1M (2013); 32.3M (2014); 21.3M (2015); 23.7M (2016) | 1D–15Y | Blood/CSF/joint fluid | 1,082 | 2011–2016 | Yes | PCV13 | Yes | 6 |
| Massora et al. 2019 (25) | Cross-sectional and surveillance | Mozambique | NR | 0–59M | Blood/CSF | 30 | 2014–2015 | No | PHiD-CV | Yes | 2 |
| Metcalf et al. 2020 (26) | Surveillance | USA | NR | <5Y | NR | 105 | 2018 | Yes | PCV13 | Yes | 8 |
| Nakano et al. 2020 (27) | Surveillance | Japan | NR | NR | Sterile/non-sterile site | 498 | 2015–2017 | Yes | PCV13 | Yes | 4 |
| Park et al. 2019 (28) ^5^ | Cross-sectional | South Korea | NR | NR | Blood/CSF/pleural fluid/abscess/tissue/urine | 48 | May 2014–May 2016 | Yes | PHiD-CV/ PCV13 | Yes | 2 |
| Picazo et al. 2019 (29) ^6^ | Surveillance | Spain | NR | NR | Sterile site | 427 | May 2007–April 2016 | Yes | PCV13 | No | NA |
| Soeters et al. 2019 (30) | Cross-sectional | Burkina Faso | NR | NR | CSF | 199 | 2014–2017 | No | PCV13 | Yes | 4 |
| Ubukata et al. 2018 (31) | Surveillance | Japan | NR | NR | Sterile site | 349 | April 2014–March 2017 | Yes | PCV13 | Yes | 4 |
| Varghese et al. 2020 (32) | Surveillance | USA | NR | NR | Sterile site | 431 | 2015–2017 | Yes | PCV13 | Yes | 7 |
| Hernandez-Bou et al. 2018 (33) | Retrospective cohort | Spain | 12.5M | 8.7–19.4M | Blood | 45 | 2011–2015 | Yes | PCV13 | No | NA |
| Hammitt et al. 2019 (34) | Surveillance | Kenya | 20M | 6–38M | Blood/CSF/NP | 34 | 2012–2016 | No | PHiD-CV | Yes | 6 |
| Lu et al. 2019 (35) | Before-after study | Taiwan | NR | NR | Sterile site | 1,408 | 2008–2017 | Yes | PHiD-CV/ PCV13 | No | NA |
| Lee et al. 2019 (36) | Retrospective cohort | Taiwan | NR | NR | Blood | 46 | 2010–2016 | Yes | PCV13 | Yes | 1 |
| Ciruela et al. 2018 (37) | Retrospective cohort | Spain | NR | NR | Blood/CSF/pleural fluid | 1,320 | 2006–2014 | Yes | PCV13 | No | NA |
| Kent et al. 2019 (38) | Surveillance | UK | 165D | 0–1Y | CSF/joint fluid/pleural fluid | 455 | 2013–2016 | Yes | PCV13 | Yes | 6 |
| Makwana et al. 2018 (39) | Surveillance | UK | NR | NR | Blood/CSF/pleural fluid | 1,059 | April 2010–March 2016 | Yes | PCV13 | Yes | 6 |
| Turner et al. 2020 (40) | Cross-sectional | Cambodia | 1.6Y | 0.9–2.3Y | Blood/CSF/pleural fluid | 1,220 | 2012–2018 | No | PCV13 | Yes | 3 |
| Richter et al. 2019 (41) | Before-after study | Austria | NR | NR | Sterile site | 36 | 2009–2017 | No | PHiD-CV | Yes | 5 |
| Silva-Costa et al. 2019 (42) | Surveillance | Portugal | NR | 0–17Y | Blood/CSF/pleural fluid | 196 | July 2012–June 2015 | Yes | PCV13 | No | NA |
| Berger et al. 2019 (43) | Retrospective cohort | Israel | 21M | 0.7–6.1M | Blood | 110 | 2007–2015 | Yes | PCV13 | Yes | 6 |
| Al-Jardani et al. 2019 (44) | Surveillance | Oman | NR | NR | Blood/CSF | 35 | June 2014–June 2016 | Yes | PCV13 | Yes | 4 |
| Diaz-Conradi et al. 2019 (45) | Prospective cohort | Spain | NR | <18Y | Blood/CSF/joint fluid/pleural fluid | 31 | January 2012–June 2016 | Yes | PCV13 | Yes | 1 |
| *AOM* |  |  |  |  |  |  |  |  |  | ***100%*** |  |
| *n=8* |  |  |  |  |  | ***731*** |  | ***n=6*** |  | ***n=8*** |  |
| Marchisio et al. 2017 (46) ^7^ | Prospective cohort | Italy | NR | <5Y | MEF | 38 | April 2015–March 2016 | Yes | PCV13 | Yes | 5 |
| Setchanova et al. 2020 (47) | Cross-sectional | Bulgaria | NR | NR | MEF | 27 | May 2012–April 2017 | No | PHiD-CV | Yes | 7 |
| Hays et al. 2017 (48) | Cross-sectional | France | NR | 0–5Y | MEF | 56 | 2013–2015 | Yes | PCV13 | Yes | 5 |
| Quirk et al. 2018 (49) | Cross-sectional | Iceland | 4.1Y | NR | MEF | 402 | 2009–2017 | No | PHiD-CV | Yes | 6 |
| Levy et al. 2019 (50) | Prospective cohort | France | 20.8M | NR | MEF | 34 | October 2015–January 2018 | Yes | PCV13 | Yes | 8 |
| Ziv et al. 2019 (51) | Retrospective cohort | Israel | NR | 0–8W | MEF | 20 | 2005–2014 | Yes | PCV13 | Yes | 4 |
| Koutouzis et al. 2016 (52) ^8^ | Cross-sectional | Greece | 25M | 12–48M | Blood/MEF | 46 | 2011–2014 | Yes | PHiD-CV/ PCV13 | Yes | 6 |
| Ubukata et al. 2018 (53) | Cross-sectional | Japan | NR | NR | MEF | 108 | June 2016–January 2017 | Yes | PCV13 | Yes | 3 |

| *CAP* |  |  |  |  |  |  |  |  |  | *100%* |  |
| --- | --- | --- | --- | --- | --- | --- | --- | --- | --- | --- | --- |
| *n=3* |  |  |  |  |  | ***235*** |  | ***n=2*** |  | ***n=3*** |  |
| Negash et al. 2019 (54) | Prospective cohort | Ethiopia | 9M | 3–18M | Blood/NP | 78 | September 2016–August 2017 | No | PHiD-CV | Yes | 6 |
| Takeuchi et al. 2020 (55) | Surveillance | Japan | NR | NR | Blood/sputum | 65 | April 2016–March 2019 | Yes | PCV13 | Yes | 6 |
| Ouldali et al. 2019 (56) | Interrupted time series | France | NR | 1.4–50Y | Blood/pleural fluid | 92 | 2009–2017 | Yes | PCV13 | Yes | 7 |

* Studies included in the primary post-PHiD-CV/-PCV13 analysis.

^1^ The study included patients aged <2–≥80 years (median 66 years). The median age for children was not reported. In total, 994 samples were serotyped in children <5 years.

^2^ Excluded from stratified analyses per PCV product due to data inconsistencies.

^3^ >90% of the samples were from sterile sites.

^4^ The study included patients aged <2–≥65 years (median not reported). In total, 331 samples were serotyped in children <5 years.

^5^ 94.5% of the samples were from sterile sites. Excluded from stratified analyses per PCV product since no separate data were available.

^6^ Not included in primary analysis, since in the Community of Madrid, PCV13 was included in the regional immunization program in 2010, was excluded in 2012, and was reintroduced in 2015.

^7^ The study included patients aged 0–60 years (median not reported). In total, 38 samples were serotyped in children <5 years.

^8^ Excluded from stratified analyses per PCV product since no separate data were available.

AOM, acute otitis media; CAP, community-acquired pneumonia; CSF, cerebrospinal fluid; D, days; IPD, invasive pneumococcal disease; M, months; MEF, middle ear fluid; N, total number of serotyped isolates; n, number of studies; NA, not applicable; NIP, national immunization program; NP, nasopharyngeal; NR, not reported; PCV, pneumococcal conjugate vaccine; PCV7, 7-valent PCV; PCV13, 13-valent PCV; PHiD-CV, pneumococcal non-typeable *Haemophilus influenzae* protein D conjugate vaccine; ref, reference; UK, United Kingdom; USA, United States of America; W, weeks; Y, years.

**Table S5.** Serotype distribution in IPD among children ≤5 years of age post-PHiD-CV/PCV13 implementation through infant national immunization programs (n=32)

| **Serotype** | **Samples identified^#^** | **Total samples serotyped^*^** | **Pooled**  **percentage**  **average^†^** | **Number of**  **studies** |
| --- | --- | --- | --- | --- |
| **12F** | 814 | 9,192 | 8.9 | 22 |
| **24F** | 703 | 8,193 | 8.6 | 18 |
| **19A** | 780 | 11,274 | 6.9 | 32 |
| **6A** | 169 | 2,822 | 6.0 | 14 |
| **33F** | 431 | 7,816 | 5.5 | 17 |
| **1** | 404 | 7,498 | 5.4 | 23 |
| **10A** | 487 | 9,835 | 5.0 | 24 |
| **3** | 541 | 11,129 | 4.9 | 30 |
| **15A** | 427 | 9,210 | 4.6 | 20 |
| **22F** | 410 | 9,251 | 4.4 | 21 |
| **23B** | 371 | 9,075 | 4.1 | 20 |
| **6B** | 164 | 4,229 | 3.9 | 18 |
| **19F** | 337 | 9,183 | 3.7 | 26 |
| **8** | 279 | 7,925 | 3.5 | 17 |
| **15B** | 85 | 2,616 | 3.2 | 12 |
| **23A** | 210 | 7,922 | 2.7 | 20 |
| **34** | 86 | 3,367 | 2.6 | 11 |
| **35B** | 233 | 9,153 | 2.5 | 23 |
| **7F** | 182 | 7,204 | 2.5 | 19 |
| **15C** | 61 | 2,689 | 2.3 | 8 |
| **38** | 192 | 8,472 | 2.3 | 20 |
| **23F** | 156 | 7,400 | 2.1 | 23 |
| **9N** | 159 | 7,785 | 2.0 | 15 |
| **11A** | 147 | 7,205 | 2.0 | 16 |
| **14** | 112 | 5,861 | 1.9 | 21 |
| **24B** | 57 | 3,625 | 1.6 | 7 |
| **21** | 60 | 4,163 | 1.4 | 9 |
| **5** | 54 | 3,761 | 1.4 | 13 |
| **13** | 30 | 2,112 | 1.4 | 9 |
| **35F** | 78 | 5,565 | 1.4 | 11 |
| **6C** | 98 | 7,134 | 1.4 | 15 |
| **16F** | 102 | 7,824 | 1.3 | 18 |
| **7C** | 21 | 2,402 | 0.9 | 7 |
| **17F** | 31 | 4,075 | 0.8 | 8 |
| **9V** | 18 | 2,917 | 0.6 | 13 |
| **18C** | 32 | 5,516 | 0.6 | 17 |
| **20** | 20 | 3,572 | 0.6 | 8 |
| **31** | 20 | 4,723 | 0.4 | 9 |
| **4** | 13 | 3,520 | 0.4 | 15 |

^#^ Total number of samples that were identified with the corresponding serotype across all included studies. References: (8-16, 18-28, 30-32, 34, 36, 38-41, 43-45).

^*^ Total number of samples that were serotyped across all studies that reported the corresponding serotype.

^†^ A pooled percentage average was calculated for each serotype by dividing ‘Samples identified’ by ‘Total samples serotyped’, multiplied by 100.

IPD, invasive pneumococcal disease; n, number of studies included in analysis; PCV13, 13-valent pneumococcal conjugate vaccine; PHiD-CV, pneumococcal non-typeable *Haemophilus influenzae* protein D conjugate vaccine.

**Table S6.** Serotype distribution in AOM among children ≤5 years of age post-PHiD-CV/PCV13 implementation through infant national immunization programs (n=8)

| Serotype | Samples identified^#^ | Total samples serotyped^*^ | Pooled percentage average^†^ | Number of studies |
| --- | --- | --- | --- | --- |
| 19F | 84 | 707 | 11.9 | 8 |
| 3 | 60 | 707 | 8.5 | 8 |
| 19A | 45 | 649 | 6.9 | 6 |
| 23A | 32 | 638 | 5.0 | 5 |
| 11A | 30 | 622 | 4.8 | 5 |
| 35B | 28 | 638 | 4.4 | 5 |
| 23B | 24 | 604 | 4.0 | 5 |
| 21 | 17 | 604 | 2.8 | 5 |

^#^ Total number of samples that were identified with the corresponding serotype across all included studies. References: (46-53).

^*^ Total number of samples that were serotyped across all studies that reported the corresponding serotype.

^†^ A pooled percentage average was calculated for each serotype by dividing ‘Samples identified’ by ‘Total samples serotyped’, multiplied by 100.

AOM, acute otitis media; n, number of studies included in analysis; PCV13, 13-valent pneumococcal conjugate vaccine; PHiD-CV, pneumococcal non-typeable *Haemophilus influenzae* protein D conjugate vaccine.

**Table S7.** Serotype distribution in CAP among children ≤5 years of age post-PHiD-CV/PCV13 implementation through infant national immunization programs (n=3)

| Serotype | Samples identified^#^ | Total samples serotyped^*^ | Pooled percentage average^†^ | Number of studies |
| --- | --- | --- | --- | --- |
| 19A | 26 | 143 | 18.2 | 2 |
| 1 | 39 | 235 | 16.6 | 3 |
| 7F | 4 | 65 | 6.2 | 1 |
| 6A | 4 | 78 | 5.1 | 1 |
| 35B | 12 | 235 | 5.1 | 3 |
| 16F | 8 | 170 | 4.7 | 2 |
| 22F | 3 | 65 | 4.6 | 1 |
| 23B | 3 | 65 | 4.6 | 1 |
| 8 | 4 | 92 | 4.3 | 1 |
| 11A | 10 | 235 | 4.3 | 3 |
| 35F | 3 | 78 | 3.8 | 1 |
| 19F | 3 | 78 | 3.8 | 1 |
| 15A | 9 | 235 | 3.8 | 3 |
| 24B | 6 | 157 | 3.8 | 2 |
| 6C | 5 | 143 | 3.5 | 2 |
| 24F | 8 | 235 | 3.4 | 3 |
| 3 | 2 | 65 | 3.1 | 1 |
| 15B | 4 | 143 | 2.8 | 2 |
| 21 | 4 | 143 | 2.8 | 2 |
| 38 | 4 | 143 | 2.8 | 2 |
| 35A | 2 | 78 | 2.6 | 1 |
| 12F | 4 | 170 | 2.4 | 2 |
| 34 | 3 | 143 | 2.1 | 2 |
| 10A | 4 | 235 | 1.7 | 3 |
| 23A | 4 | 235 | 1.7 | 3 |
| 37 | 1 | 65 | 1.5 | 1 |
| 11B | 1 | 78 | 1.3 | 1 |
| 13 | 1 | 78 | 1.3 | 1 |
| 18A | 1 | 78 | 1.3 | 1 |
| 20 | 1 | 78 | 1.3 | 1 |
| 33B | 1 | 78 | 1.3 | 1 |
| 9L | 1 | 78 | 1.3 | 1 |
| 14 | 1 | 78 | 1.3 | 1 |
| 33F | 2 | 157 | 1.3 | 2 |
| 15C | 2 | 157 | 1.3 | 2 |
| 7B | 1 | 92 | 1.1 | 1 |
| 9N | 1 | 92 | 1.1 | 1 |

^#^ Total number of samples that were identified with the corresponding serotype across all included studies. References: (54-56).

^*^ Total number of samples that were serotyped across all studies that reported the corresponding serotype.

^†^ A pooled percentage average was calculated for each serotype by dividing ‘Samples identified’ by ‘Total samples serotyped’, multiplied by 100.

CAP, community-acquired pneumonia; n, number of studies included in analysis; PCV13, 13-valent pneumococcal conjugate vaccine; PHiD-CV, pneumococcal non-typeable *Haemophilus influenzae* protein D conjugate vaccine.

**Figure S1.** Serotype distribution in IPD among children ≤5 years of age post-PHiD-CV/PCV13 uptake in infants (either through infant national immunization programs or private markets) (n=38)


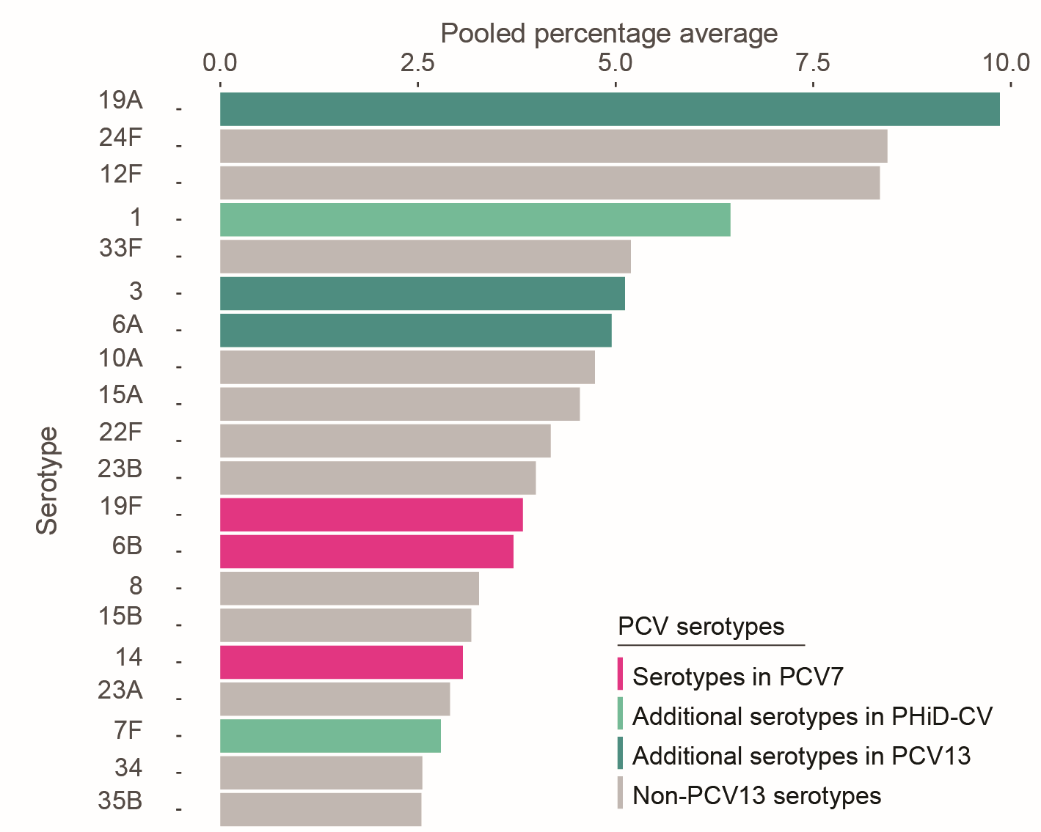


Note: The top 20 serotypes are shown. Serotypes are represented by colors corresponding to the lowest valency PCV product in which they are included. In the PCV legend, the additional serotypes included in the product are relative to the next lower valency product. Pooled percentage averages were calculated for each serotype individually, thus the sum of all serotypes may exceed 100%. Serotype-specific pooled percentage averages were calculated only if 5 or more studies reported on the respective serotype.

IPD, invasive pneumococcal disease; n, number of studies included in analysis; PCV, pneumococcal conjugate vaccine; PCV7, 7-valent PCV; PCV13, 13-valent PCV; PHiD-CV, pneumococcal non-typeable *Haemophilus influenzae* protein D conjugate vaccine.

Figure S2. Serotype distribution in IPD among children ≤5 years of age post-PCV7 implementation through infant national immunization programs (n=9)


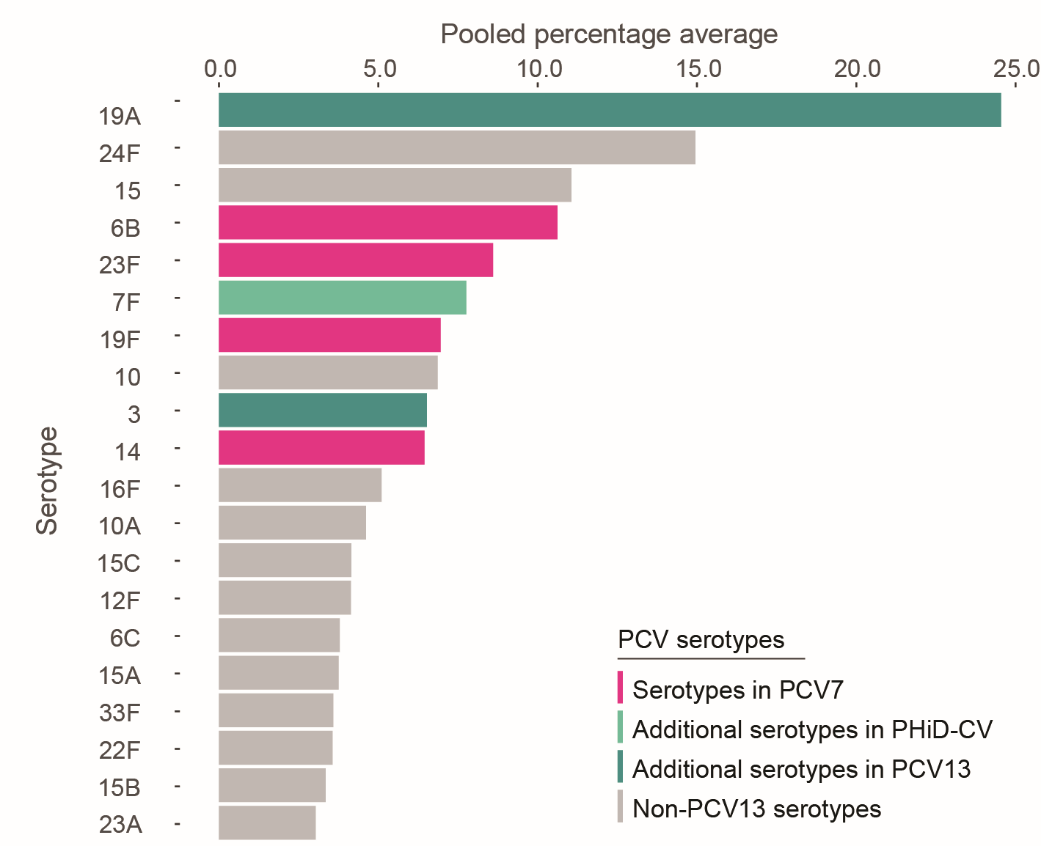


Note: The top 20 serotypes are shown. Serotypes are represented by colors corresponding to the lowest valency PCV product in which they are included. In the PCV legend, the additional serotypes included in the product are relative to the next lower valency product. Pooled percentage averages were calculated for each serotype individually, thus the sum of all serotypes may exceed 100%. Serotype-specific pooled percentage averages were calculated only if 5 or more studies reported on the respective serotype.

IPD, invasive pneumococcal disease; n, number of studies included in analysis; PCV, pneumococcal conjugate vaccine; PCV7, 7-valent PCV; PCV13, 13-valent PCV; PHiD-CV, pneumococcal non-typeable *Haemophilus influenzae* protein D conjugate vaccine.

**Figure S3.** Serotype distribution in AOM among children ≤5 years of age post-PCV7 implementation through infant national immunization programs (n=15)


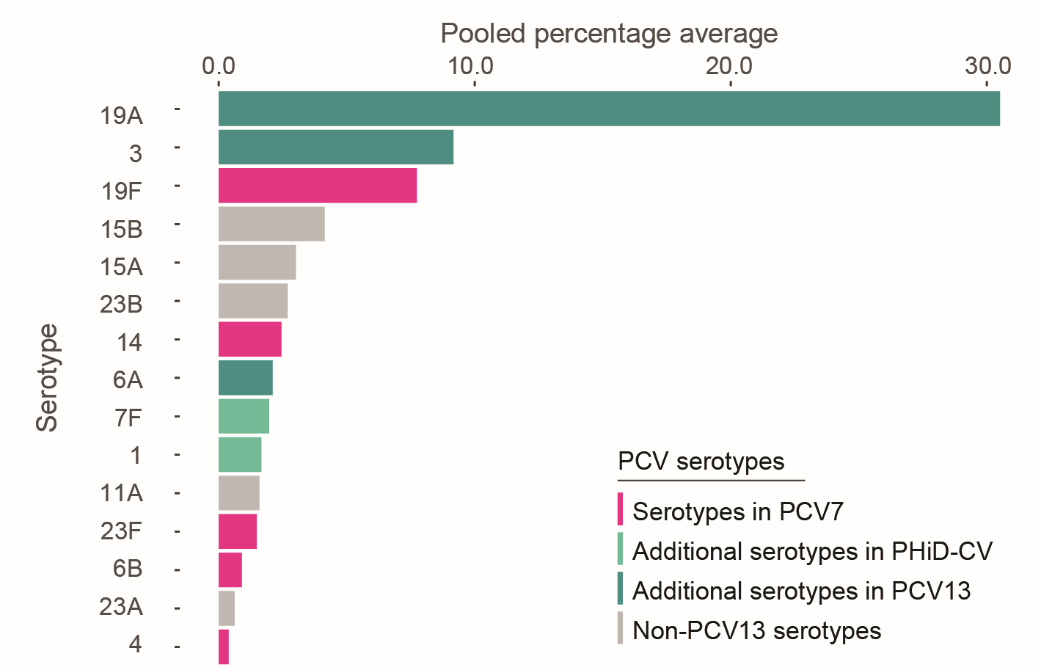


Note: Serotypes are represented by colors corresponding to the lowest valency PCV product in which they are included. In the PCV legend, the additional serotypes included in the product are relative to the next lower valency product. Pooled percentage averages were calculated for each serotype individually, thus the sum of all serotypes may exceed 100%. Serotype-specific pooled percentage averages were calculated only if 5 or more studies reported on the respective serotype. One study with sample type of unknown origin was included, as they were reported as “acute otitis media samples non-invasive disease”.

AOM, acute otitis media; n, number of studies included in analysis; PCV, pneumococcal conjugate vaccine; PCV7, 7-valent PCV; PCV13, 13-valent PCV; PHiD-CV, pneumococcal non-typeable *Haemophilus influenzae* protein D conjugate vaccine.

**Figure S4.** Serotype distribution in CAP among children ≤5 years of age post-PHiD-CV uptake in infants (either through infant national immunization programs or private markets) (n=1)


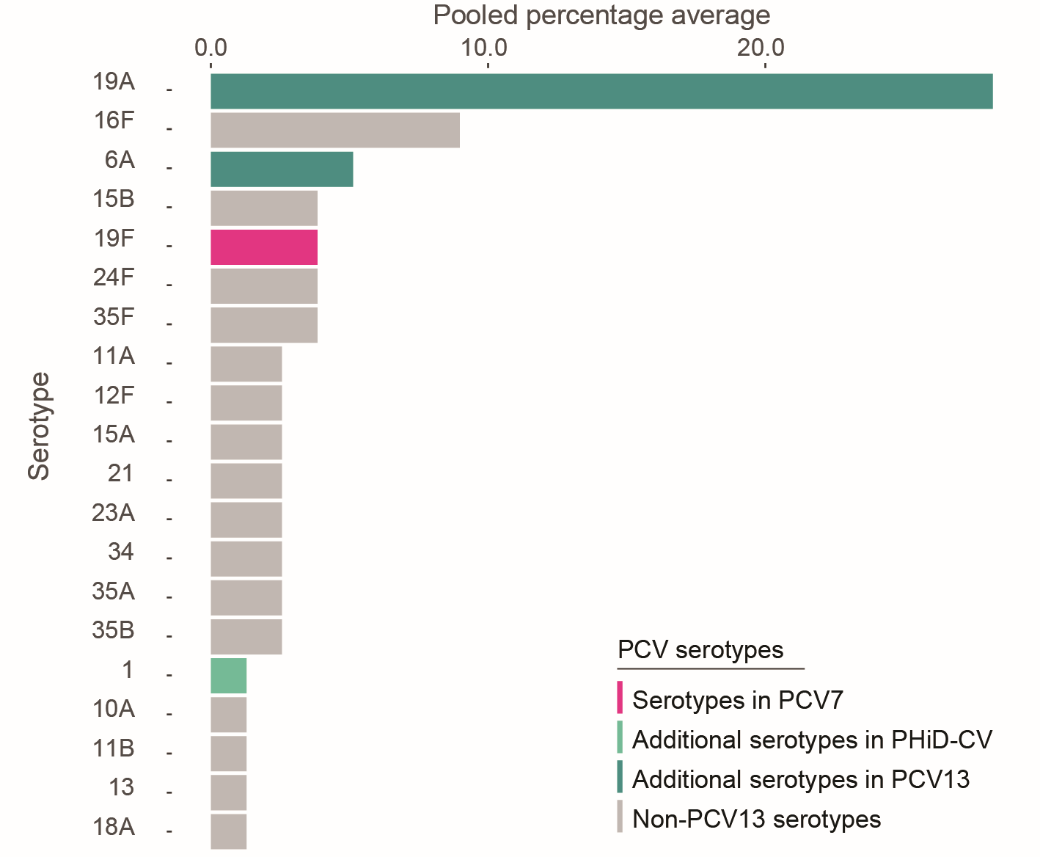


Note: The top 20 serotypes are shown. Serotypes are represented by colors corresponding to the lowest valency PCV product in which they are included. In the PCV legend, the additional serotypes included in the product are relative to the next lower valency product. Pooled percentage averages were calculated for each serotype individually, thus the sum of all serotypes may exceed 100%.

CAP, community-acquired pneumonia; n, number of studies included in analysis; PCV, pneumococcal conjugate vaccine; PCV7, 7-valent PCV; PCV13, 13-valent PCV; PHiD-CV, pneumococcal non-typeable *Haemophilus influenzae* protein D conjugate vaccine.

**Figure S5.** Serotype distribution in CAP among children ≤5 years of age post-PCV13 uptake in infants (either through infant national immunization programs or private markets) (n=2)


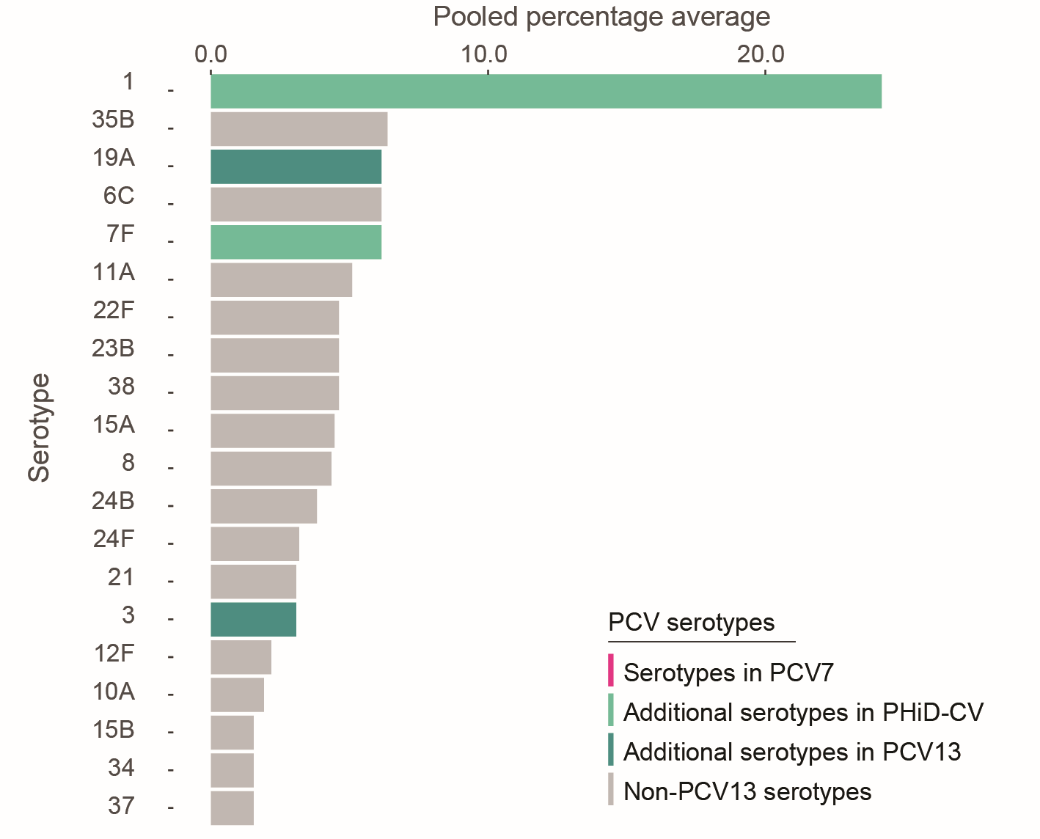


Note: The top 20 serotypes are shown. Serotypes are represented by colors corresponding to the lowest valency PCV product in which they are included. In the PCV legend, the additional serotypes included in the product are relative to the next lower valency product. Pooled percentage averages were calculated for each serotype individually, thus the sum of all serotypes may exceed 100%. Serotype-specific pooled percentage averages were calculated irrespective of the number of studies reporting on it.

CAP, community-acquired pneumonia; n, number of studies included in analysis; PCV, pneumococcal conjugate vaccine; PCV7, 7-valent PCV; PCV13, 13-valent PCV; PHiD-CV, pneumococcal non-typeable *Haemophilus influenzae* protein D conjugate vaccine.

**Figure S6.** Serotype distribution in CAP among children ≤5 years of age post-PCV7 implementation through infant national immunization programs (n=5)


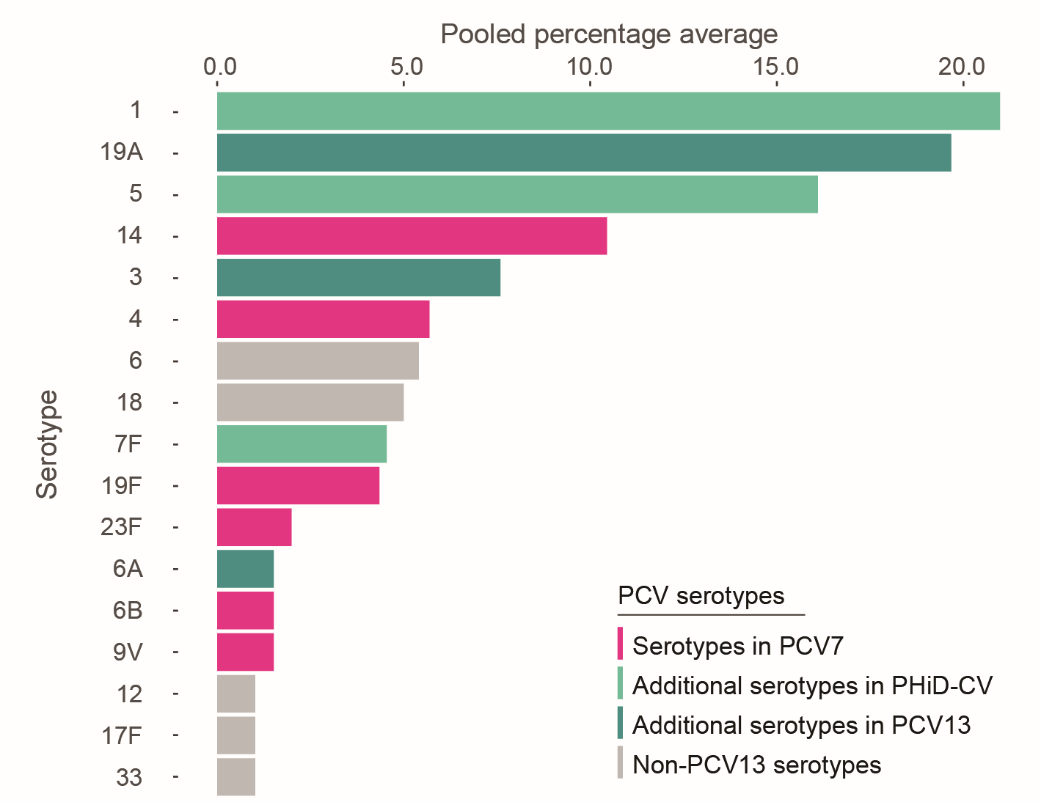


Note: The top 20 serotypes are shown. Serotypes are represented by colors corresponding to the lowest valency PCV product in which they are included. In the PCV legend, the additional serotypes included in the product are relative to the next lower valency product. Pooled percentage averages were calculated for each serotype individually, thus the sum of all serotypes may exceed 100%. Serotype-specific pooled percentage averages were calculated irrespective of the number of studies reporting on it.

CAP, community-acquired pneumonia; n, number of studies included in analysis; PCV, pneumococcal conjugate vaccine; PCV7, 7-valent PCV; PCV13, 13-valent PCV; PHiD-CV, pneumococcal non-typeable *Haemophilus influenzae* protein D conjugate vaccine.

# References

1. Randle E, Ninis N, Inwald D. Invasive pneumococcal disease. Arch Dis Child Educ Pract Ed (2011) 96:183-90. doi: 10.1136/adc.2010.191718

2. National Institute for Health and Care Excellence. Clinical Knowledge Summaries (CKS) acute otitis media (2021). <https://cks.nice.org.uk/topics/otitis-media-acute/>. [Accessed April 14, 2025].

3. Lieberthal AS, Carroll AE, Chonmaitree T, Ganiats TG, Hoberman A, Jackson MA, et al. The diagnosis and management of acute otitis media. Pediatrics (2013) 131:e964-e99. doi: 10.1542/peds.2012-3488

4. National Institute for Health and Care Excellence. Pneumonia: diagnosis and management of community- and hospital-acquired pneumonia in adults (2012). <https://www.nice.org.uk/guidance/cg191/documents/pneumonia-final-scope2>. [Accessed April 14, 2025].

5. DistillerSR. <https://www.distillersr.com/>. [Accessed April 14, 2025].

6. Automeris LLC. WebPlotDigitizer. <https://automeris.io/WebPlotDigitizer>. [Accessed April 14, 2025].

7. International Vaccine Access Center (IVAC), Johns Hopkins Bloomberg School of Public Health. VIEW-hub. <https://view-hub.org/vaccine/pcv>. [Accessed April 14, 2025].

8. Amin-Chowdhury Z, Collins S, Sheppard C, Litt D, Fry NK, Andrews N, et al. Characteristics of invasive pneumococcal disease caused by emerging serotypes after the introduction of the 13-valent pneumococcal conjugate vaccine in England: A prospective observational cohort study, 2014-2018. Clin Infect Dis (2020) 71:e235-e43. doi: 10.1093/cid/ciaa043

9. Baxter R, Aukes L, Pelton SI, Yee A, Klein NP, Gruber WC, et al. Impact of the 13-valent pneumococcal conjugate vaccine on invasive pneumococcal disease after introduction into routine pediatric use. J Pediatric Infect Dis Soc (2021) 10:141-50. doi: 10.1093/jpids/piaa035

10. Berezin EN, Jarovsky D, Cardoso MRA, Mantese OC. Invasive pneumococcal disease among hospitalized children in Brazil before and after the introduction of a pneumococcal conjugate vaccine. Vaccine (2020) 38:1740-5. doi: 10.1016/j.vaccine.2019.12.038

11. Ceyhan M, Aykac K, Gurler N, Ozsurekci Y, Öksüz L, Altay AÖ, et al. Serotype distribution of Streptococcus pneumonia in children with invasive disease in Turkey: 2015-2018. Hum Vaccin Immunother (2020) 17:2352. doi: 10.1080/21645515.2020.1747931

12. Cohen R, Levy C, Ouldali N, Goldrey M, Béchet S, Bonacorsi S, et al. Invasive disease potential of pneumococcal serotypes in children after PCV13 Implementation. Clin Infect Dis (2021) 72:1453-6. doi: 10.1093/cid/ciaa917

13. Corcoran M, Mereckiene J, Cotter S, Murchan S, Cunney R, Humphreys H. Invasive Streptococcus pneumoniae infections and vaccine failures in children in Ireland from the postvaccine era from 2007 to 2018. Pediatr Infect Dis J (2020) 39:339-44. doi: 10.1097/inf.0000000000002549

14. de Miguel S, Domenech M, González-Camacho F, Sempere J, Vicioso D, Sanz JC, et al. Nationwide trends of invasive pneumococcal disease in Spain (2009-2019) in children and adults during the pneumococcal conjugate vaccine era. Clin Infect Dis (2021) 73:e3778-e87. doi: 10.1093/cid/ciaa1483

15. Desmet S, Lagrou K, Wyndham-Thomas C, Braeye T, Verhaegen J, Maes P, et al. Dynamic changes in paediatric invasive pneumococcal disease after sequential switches of conjugate vaccine in Belgium: a national retrospective observational study. Lancet Infect Dis (2021) 21:127-36. doi: 10.1016/s1473-3099(20)30173-0

16. Desmet S, Wouters I, Van Heirstraeten L, Beutels P, Van Damme P, Malhotra-Kumar S, et al. In-depth analysis of pneumococcal serotypes in Belgian children (2015–2018): Diversity, invasive disease potential, and antimicrobial susceptibility in carriage and disease. Vaccine (2021) 39:372-9. doi: 10.1016/j.vaccine.2020.11.044

17. Hernández S, Muñoz-Almagro C, Ciruela P, Soldevila N, Izquierdo C, Codina MG, et al. Invasive pneumococcal disease and influenza activity in a pediatric population: Impact of PCV13 vaccination in pandemic and nonpandemic influenza periods. J Clin Microbiol (2019) 57:e00363-19. doi: 10.1128/JCM.00363-19

18. Hernstadt H, Cheung A, Hurem D, Vasilunas N, Phuong LK, Quinn P, et al. Changing epidemiology and predisposing factors for invasive pneumococcal disease at two Australian tertiary hospitals. Pediatr Infect Dis J (2020) 39:1-6. doi: 10.1097/inf.0000000000002489

19. Iwata S, Takata M, Morozumi M, Miyairi I, Matsubara K, Ubukata K. Drastic reduction in pneumococcal meningitis in children owing to the introduction of pneumococcal conjugate vaccines: Longitudinal analysis from 2002 to 2016 in Japan. J Infect Chemother (2021) 27:604-12. doi: 10.1016/j.jiac.2020.11.019

20. Izquierdo C, Ciruela P, Hernández S, García-García JJ, Esteva C, Moraga-Llop F, et al. Pneumococcal serotypes in children, clinical presentation and antimicrobial susceptibility in the PCV13 era. Epidemiol Infect (2020) 148:e279. doi: 10.1017/s0950268820002708

21. Kambire D, Soeters HM, Ouedraogo-Traore R, Medah I, Sangare L, Yameogo I, et al. Early impact of 13-valent pneumococcal conjugate vaccine on pneumococcal meningitis-Burkina Faso, 2014-2015. J Infect (2018) 76:270-9. doi: 10.1016/j.jinf.2017.12.002

22. Kaplan SL, Barson WJ, Lin PL, Romero JR, Bradley JS, Tan TQ, et al. Invasive pneumococcal disease in children's hospitals: 2014-2017. Pediatrics (2019) 144:e20190567. doi: 10.1542/peds.2019-0567

23. Ladhani SN, Collins S, Djennad A, Sheppard CL, Borrow R, Fry NK, et al. Rapid increase in non-vaccine serotypes causing invasive pneumococcal disease in England and Wales, 2000–17: a prospective national observational cohort study. Lancet Infect Dis (2018) 18:441-51. doi: 10.1016/S1473-3099(18)30052-5

24. Levy C, Varon E, Ouldali N, Béchet S, Bonacorsi S, Cohen R. Changes in invasive pneumococcal disease spectrum after 13-valent pneumococcal conjugate vaccine implementation. Clin Infect Dis (2020) 70:446-54. doi: 10.1093/cid/ciz221

25. Massora S, Lessa FC, Moiane B, Pimenta FC, Mucavele H, Chaúque A, et al. Invasive disease potential of Streptococcus pneumoniae serotypes before and after 10-valent pneumococcal conjugate vaccine introduction in a rural area, southern Mozambique. Vaccine (2019) 37:7470-7. doi: 10.1016/j.vaccine.2019.09.079

26. Metcalf BJ, Chochua S, Walker H, Tran T, Li Z, Varghese J, et al. Invasive pneumococcal strain distributions and isolate clusters associated with persons experiencing homelessness during 2018. Clinical infectious diseases (2020) 72:e948–e56. doi: 10.1093/cid/ciaa1680

27. Nakano S, Fujisawa T, Ito Y, Chang B, Matsumura Y, Yamamoto M, et al. Nationwide surveillance of paediatric invasive and non-invasive pneumococcal disease in Japan after the introduction of the 13-valent conjugated vaccine, 2015-2017. Vaccine (2020) 38:1818-24. doi: 10.1016/j.vaccine.2019.12.022

28. Park DC, Kim SH, Yong D, Suh IB, Kim YR, Yi J, et al. Serotype distribution and antimicrobial resistance of invasive and noninvasive Streptococcus pneumoniae isolates in Korea between 2014 and 2016. Ann Lab Med (2019) 39:537-44. doi: 10.3343/alm.2019.39.6.537

29. Picazo JJ, Ruiz-Contreras J, Casado-Flores J, Negreira S, Baquero-Artigao F, Hernández-Sampelayo T, et al. Impact of 13-valent pneumococcal conjugate vaccination on invasive pneumococcal disease in children under 15 years old in Madrid, Spain, 2007 to 2016: The HERACLES clinical surveillance study. Vaccine (2019) 37:2200-7. doi: 10.1016/j.vaccine.2019.03.015

30. Soeters HM, Kambiré D, Sawadogo G, Ouédraogo-Traoré R, Bicaba B, Medah I, et al. Impact of 13-valent pneumococcal conjugate vaccine on pneumococcal meningitis, Burkina Faso, 2016-2017. J Infect Dis (2019) 220:S253-S62. doi: 10.1093/infdis/jiz301

31. Ubukata K, Takata M, Morozumi M, Chiba N, Wajima T, Hanada S, et al. Effects of pneumococcal conjugate vaccine on genotypic penicillin resistance and serotype changes, Japan, 2010-2017. Emerg Infect Dis (2018) 24:2010-20. doi: 10.3201/eid2411.180326

32. Varghese J, Chochua S, Tran T, Walker H, Li Z, Snippes Vagnone PM, et al. Multistate population and whole genome sequence-based strain surveillance of invasive pneumococci recovered in the USA during 2017. Clin Microbiol Infect (2020) 26:512.e1-.e10. doi: 10.1016/j.cmi.2019.09.008

33. Hernández-Bou S, Gomez B, Mintegi S, Garcia-Garcia JJ, Diseases. BSWGotI, Emergencies. WGotSSoP. Occult bacteremia etiology following the introduction of 13-valent pneumococcal conjugate vaccine: a multicenter study in Spain. Eur J Clin Microbiol Infect Dis (2018) 37:1449-55. doi: 10.1007/s10096-018-3270-2

34. Hammitt LL, Etyang AO, Morpeth SC, Ojal J, Mutuku A, Mturi N, et al. Effect of ten-valent pneumococcal conjugate vaccine on invasive pneumococcal disease and nasopharyngeal carriage in Kenya: a longitudinal surveillance study. Lancet (2019) 393:2146-54. doi: 10.1016/s0140-6736(18)33005-8

35. Lu C-Y, Chiang C-S, Chiu C-H, Wang ET, Chen YY, Yao SM, et al. Successful control of Streptococcus pneumoniae 19A replacement with a catch-up primary vaccination program in Taiwan. Clin Infect Dis (2019) 69:1581-7. doi: 10.1093/cid/ciy1127

36. Lee M-C, Kuo K-C. The clinical implication of serotype distribution and drug resistance of invasive pneumococcal disease in children: A single center study in southern Taiwan during 2010-2016. J Microbiol Immunol Infect (2019) 52:937-46. doi: 10.1016/j.jmii.2019.04.006

37. Ciruela P, Izquierdo C, Broner S, Munoz-Almagro C, Hernandez S, Ardanuy C, et al. The changing epidemiology of invasive pneumococcal disease after PCV13 vaccination in a country with intermediate vaccination coverage. Vaccine (2018) 36:7744-52. doi: 10.1016/j.vaccine.2018.05.026

38. Kent A, Makwana A, Sheppard CL, Collins S, Fry NK, Heath PT, et al. Invasive pneumococcal disease in UK children <1 year of age in the post-13-valent pneumococcal conjugate vaccine era: what are the risks now? Clin Infect Dis (2019) 69:84-90. doi: 10.1093/cid/ciy842

39. Makwana A, Sheppard C, Borrow R, Fry N, Andrews NJ, Ladhani SN. Characteristics of children with invasive pneumococcal disease after the introduction of the 13-valent pneumococcal conjugate vaccine in England and Wales, 2010-2016. Pediatr Infect Dis J (2018) 37:697-703. doi: 10.1097/INF.0000000000001845

40. Turner P, Leab P, Ly S, Sao S, Miliya T, Heffelfinger JD, et al. Impact of 13-valent pneumococcal conjugate vaccine on colonization and invasive disease in Cambodian children. Clin Infect Dis (2020) 70:1580-8. doi: 10.1093/cid/ciz481

41. Richter L, Schmid D, Kanitz EE, Zwazl I, Pollabauer E, Jasinska J, et al. Invasive pneumococcal diseases in children and adults before and after introduction of the 10-valent pneumococcal conjugate vaccine into the Austrian national immunization program. PloS One (2019) 14:e0210081. doi: 10.1371/journal.pone.0210081

42. Silva-Costa C, Brito MJ, Aguiar SI, Lopes JP, Ramirez M, Melo-Cristino J, et al. Dominance of vaccine serotypes in pediatric invasive pneumococcal infections in Portugal (2012-2015). Sci Rep (2019) 9:6. doi: 10.1038/s41598-018-36799-x

43. Berger Y, Adler A, Ariel T, Rokney A, Averbuch D, Grisaru-Soen G. Paediatric community-acquired bacteraemia, pneumococcal invasive disease and antibiotic resistance fell after the pneumococcal conjugate vaccine was introduced. Acta Paediatr (2019) 108:1321-8. doi: 10.1111/apa.14670

44. Al-Jardani A, Al Rashdi A, Al Jaaidi A, Al Bulushi M, Al Mahrouqi S, Al-Abri S, et al. Serotype distribution and antibiotic resistance among invasive Streptococcus pneumoniae from Oman post 13-valent vaccine introduction. Int J Infect Dis (2019) 85:135-40. doi: 10.1016/j.ijid.2019.05.027

45. Díaz-Conradi A, Hernández S, García-García JJ, Munoz-Almagro C, Moraga-Llop F, Ciruela P, et al. Complicated pneumococcal pneumonia with pleural effusion or empyema in the 13-valent pneumococcal conjugate vaccine era. Pediatr Pulmonol (2019) 54:517-24. doi: 10.1002/ppul.24279

46. Marchisio P, Esposito S, Picca M, Baggi E, Terranova L, Orenti A, et al. Serotypes not included in 13-valent pneumococcal vaccine as causes of acute otitis media with spontaneous tympanic membrane perforation in a geographic area with high vaccination coverage. Pediatr Infect Dis J (2017) 36:521-3. doi: 10.1097/INF.0000000000001485

47. Setchanova L, Stancheva I, Popova D, Alexandrova A, Mitov I. Bacterial spectrum of acute otitis media in Bulgarian children during the 10-valent pneumococcal conjugate vaccine era. J Pediatr Infect Dis (2020) 15:135-43. doi: 10.1055/s-0040-1701647

48. Hays C, Vermee Q, Agathine A, Dupuis A, Varon E, Poyart C, et al. Demonstration of the herd effect in adults after the implementation of pneumococcal vaccination with PCV13 in children. Eur J Clin Microbiol Infect Dis (2017) 36:831-8. doi: 10.1007/s10096-016-2868-5

49. Quirk SJ, Haraldsson G, Erlendsdottir H, Hjalmarsdottir MA, van Tonder AJ, Hrafnkelsson B, et al. Effect of vaccination on pneumococci isolated from the nasopharynx of healthy children and the middle ear of children with otitis media in Iceland. J Clin Microbiol (2018) 56:e01046-18. doi: 10.1128/jcm.01046-18

50. Levy C, Varon E, Ouldali N, Wollner A, Thollot F, Corrard F, et al. Bacterial causes of otitis media with spontaneous perforation of the tympanic membrane in the era of 13 valent pneumococcal conjugate vaccine. PloS One (2019) 14:e0211712. doi: 10.1371/journal.pone.0211712

51. Ziv O, Kraus M, Holcberg R, Dinur AB, Kordeluk S, Kaplan D, et al. Acute otitis media in infants younger than two months of age: Epidemiologic and microbiologic characteristics in the era of pneumococcal conjugate vaccines. Int J Pediatr Otorhinolaryngol (2019) 119:123-30. doi: 10.1016/j.ijporl.2019.01.031

52. Koutouzis EI, Michos A, Koutouzi FI, Chatzichristou P, Parpounas K, Georgaki A, et al. Pneumococcal mastoiditis in children before and after the introduction of conjugate pneumococcal vaccines. Pediatr Infect Dis J (2016) 35:292-6. doi: 10.1097/inf.0000000000000995

53. Ubukata K, Morozumi M, Sakuma M, Takata M, Mokuno E, Tajima T, et al. Etiology of acute otitis media and characterization of pneumococcal isolates after introduction of 13-valent pneumococcal conjugate vaccine in Japanese children. Pediatr Infect Dis J (2018) 37:598-604. doi: 10.1097/inf.0000000000001956

54. Negash AA, Asrat D, Abebe W, Hailemariam T, Gebre M, Verhaegen J, et al. Pneumococcal carriage, serotype distribution, and risk factors in children with community-acquired pneumonia, 5 years after introduction of the 10-valent pneumococcal conjugate vaccine in Ethiopia. Open Forum Infectious Diseases (2019) 6. doi: 10.1093/ofid/ofz259

55. Takeuchi N, Naito S, Ohkusu M, Abe K, Shizuno K, Takahashi Y, et al. Epidemiology of hospitalised paediatric community-acquired pneumonia and bacterial pneumonia following the introduction of 13-valent pneumococcal conjugate vaccine in the national immunisation programme in Japan. Epidemiol Infect (2020) 148:e91. doi: 10.1017/s0950268820000813

56. Ouldali N, Levy C, Minodier P, Morin L, Biscardi S, Aurel M, et al. Long-term association of 13-valent pneumococcal conjugate vaccine implementation with rates of community-acquired pneumonia in children. Jama Pediatr (2019) 173:362-70. doi: 10.1001/jamapediatrics.2018.5273
